# hUC-MSC preserves erectile function by restoring mitochondrial mass of penile smooth muscle cells in a rat model of cavernous nerve injury via SIRT1/PGC-1a/TFAM signaling

**DOI:** 10.1186/s40659-024-00578-y

**Published:** 2025-01-27

**Authors:** Mengbo Yang, Xinda Chen, Ming Zhang, Xiaolin Zhang, Dongdong Xiao, Huiming Xu, Mujun Lu

**Affiliations:** 1 Department of Urology and Andrology, Renji Hospital, Shanghai Institute of Andrology, School of Medicine, Shanghai Jiaotong University, Shanghai, 200127 China; 2 State Laboratory of Systems Medicine for Cancer, Renji-MedX Clinical Stem Cell Research Center, Ren Ji Hospital, School of Medicine, Shanghai Jiao Tong University, Shanghai, 200127 China

**Keywords:** Cavernous nerve injury-induced erectile dysfunction, Corpus cavernous smooth muscle cell, Mesenchymal stem cell, SIRT1/PGC-1α/TFAM pathway

## Abstract

**Background:**

Cavernous nerve injury-induced erectile dysfunction (CNI-ED) is a common complication following radical prostatectomy and severely affects patients’ quality of life. The mitochondrial impairment in corpus cavernosum smooth muscle cells (CCSMCs) may be an important pathological mechanism of CNI-ED. Previous studies have shown that transplantation of human adipose derived stem cells (ADSC) can alleviate CNI-ED in a rat model. However, little is known about the effect of human umbilical cord mesenchymal stem cells (hUC-MSC) on CNI-ED. It remains unclear whether hUC-MSC can ameliorate mitochondrial damage in CCSMCs. In this study, we aimed to investigate the impacts of hUC-MSC on the mitochondrial mass and function of CCSMCs, as well as elucidate its underlying molecular mechanism.

**Methods:**

The CNI-ED rat model was established by bilaterally crushing cavernous nerves. Subsequently, hUC-MSC were transplanted into the cavernosum and ADSC were injected as a positive control group. Erectile function evaluation and histological detection were performed 4 weeks after cell transplantation. In vitro, CCSMCs underwent hypoxia and were then co-cultured with ADSC or hUC-MSC using a transwell system. The mitochondrial mass and function, as well as signaling pathways, were investigated. To explore the role of the SIRT1/PGC-1α/TFAM pathway in regulating mitochondrial biogenesis of CCSMCs, we knocked down SIRT1 by siRNA.

**Results:**

The administration of hUC-MSC significantly improved erectile function of CNI-ED rats and reduced the ratio of collagen to smooth muscle. Specifically, hUC-MSC treatment restored mitochondrial mass and function in CCSMCs injured by CNI or hypoxia, and inhibited the apoptosis of CCSMCs. Mechanistically, the application of hUC-MSC activated SIRT1/PGC-1α/TFAM pathway both in rat penile tissues and CCSMCs. In addition, knockdown of SIRT1 in CCSMCs abolished the protective effects of hUC-MSC on mitochondrial mass and function, while leading to an increase in cellular apoptosis.

**Conclusions:**

hUC-MSC contribute to the recovery of erectile function in CNI-ED rats by restoring mitochondrial mass and function of CCSMCs through the SIRT1/PGC-1α/TFAM pathway. Our present study offers new insights into the role and molecular mechanisms of hUC-MSC in regulating mitochondrial homeostasis, thereby facilitating the restoration of the erectile function in CNI-ED.

**Supplementary Information:**

The online version contains supplementary material available at 10.1186/s40659-024-00578-y.

## Introduction

Cavernous nerve injury-induced erectile dysfunction (CNI-ED) is the most common complication of radical prostatectomy (RP) and severely affects the life quality of more than 70% of patients [1–3]. Despite advances in the nerve-sparing techniques, only 39%−63% of patients following RP retain their sexual potency [1]. RP damages the cavernous nerves (CNs), resulting in the loss of both erotic and nocturnal erections, reduced cavernous sinus perfusion, and subsequent hypoxia within the cavernous tissue. Ultimately, RP induces structural alterations in the corpus cavernosum, resulting in increased penile atrophy, enhanced apoptosis of corpus cavernosum smooth muscle cells (CCSMCs), and an elevated ratio of collagen to smooth muscle [4–8]. Currently, oral phosphodiesterase 5 inhibitors are widely used as first-line medications for CNI-ED, but their therapeutic effect remains constrained [4]. Therefore, investigating novel therapies for CNI-ED is required. Preclinical and clinical trials have indicated that mesenchymal stem cells (MSCs) transplantation into corpus cavernosum may represent an effective and promising strategy for the treatment of CNI-ED [9–12]. However, little is known about whether human umbilical cord mesenchymal stem cells (hUC-MSC) have a therapeutic effect on CNI-ED.

Previous studies have demonstrated that the mitochondria in penile smooth muscle cells and endothelial cells of rat models with erectile dysfunction are impaired. Furthermore, the activation of the mitochondrial apoptotic pathway in circulating angiogenic cells has been observed in patients with ED, suggesting that mitochondrial dysfunction may represent a significant pathological mechanism under the condition of ED [13, 14]. Mitochondria are important organelles responsible for supplying cellular energy, regulating cell apoptosis and calcium homoeostasis, as well as generating and scavenging of reactive oxygen species (ROS) [15, 16]. They are also highly dynamic organelles involving in the processes including mitochondrial fission, fusion, biogenesis, mitophagy and transport. These processes coordinately regulate mitochondrial morphology, quantity, quality, and inheritance [17], thereby ensuring the maintenance of mitochondrial homeostasis to meet the physiological needs of cells [18, 19]. However, hypoxia disrupts mitochondrial homeostasis, resulting in a reduction of mitochondrial mass and levels of oxidative phosphorylation (OXPHOS), while simultaneously increasing cell apoptosis [20–22]. Notably, it has been reported that MSCs can restore mitochondrial function and reduce cell apoptosis [23–27]. It is hypothesized that hUC-MSC may influence mitochondrial function of penile smooth muscle cells in rat models with erectile dysfunction.

Mitochondrial biogenesis is a complex and tightly regulated process that involves the coordinated expression of both nuclear and mitochondrial genomes, which plays an important role in maintaining mitochondrial homeostasis [18, 19]. The SIRT1/PGC-1α/TFAM signaling pathway has been reported to be involved in regulating mitochondrial biogenesis [19, 27, 28]. SIRT1, an NAD^+^-dependent protein deacetylase which deacetylates and activates PGC-1α, which serves as a master regulator of mitochondrial biogenesis. In turn, PGC-1α stimulates the transcription of downstream nuclear respiratory factor 1 (NRF1), which subsequently promotes the transcription of mitochondrial transcription factor A (TFAM). TFAM is a critical regulator for the transcription of mitochondrial DNA or RNA [29, 30]. These studies indicate that MSCs may activate the SIRT1/PGC-1α/TFAM pathway, thereby promoting mitochondrial biogenesis and repair mitochondrial function. However, it remains unclear whether SIRT1/PGC-1α/TFAM pathway is involved in regulating mitochondrial biogenesis of CCSMCs under conditions of CNI-ED.

In the present study, we first evaluated the efficacy of hUC-MSC in the CNI-ED rat model. Subsequently, we examined the protective effects of hUC-MSC on mitochondrial mass and function in CCSMCs, while also investigating the underlying molecular mechanism of hUC-MSC.

## Results

### Characterization of hUC-MSC

hUC-MSC and ADSC were isolated from the human umbilical cord and adipose tissues. Both types of cells displayed typical spindle shapes (Additional file 1: Fig. S1A) and showed high expression of CD105, CD90, and CD73, while expressing low levels of CD31, CD34, and HLA-DR (Additional file 1: Fig. S1B). Additionally, they exhibited adipogenic and osteogenic potentials (Additional file 1: Fig. S1C and Fig. S1D). These data collectively indicate that the isolated hUC-MSC and ADSC are highly pure MSCs with strong differentiation capabilities suitable for the subsequent experiments.

### hUC-MSC treatment restores erectile function in CNI-ED rats

**Fig. 1:**
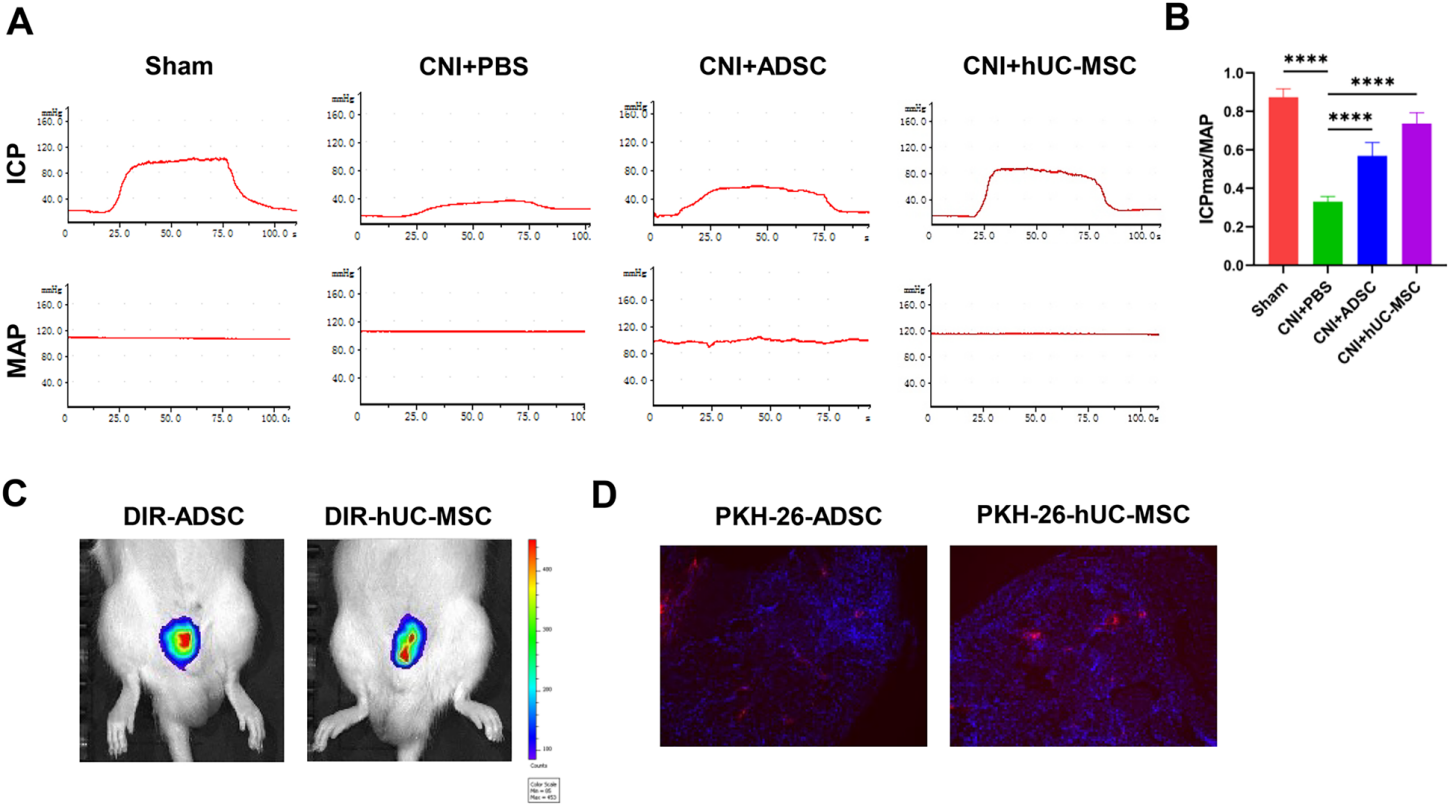
ADSC or hUC-MSC transplantation restores erectile function in CNI-ED rats. **A** Representative images of the ICP and MAP in each group. **B** Quantification of maximal ICP to MAP ratio (ICPmax/MAP) of different groups. Data are presented as mean ± SD (n = 6). ****P < 0.0001). **C** Representative DIR dye-labelled ADSC or hUC-MSC images in corpora cavernosa 24 h after injection. **D** Representative images of PKH26 dye labeled-ADSC or hUC-MSC in corpora cavernosa 72 h after injection

To investigate the impact of hUC-MSC on ED, a CNI-ED rat model was established as previously described [31]. hUC-MSC were injected into cavernous body of the rats, with ADSC used as a positive control. Erectile function was evaluated using ICP and ICP_max_/MAP at 4 weeks after cell transplantation. the ICP and ICP_max_/MAP of rats in PBS group showed a significant decrease compared to the sham group; however, hUC-MSC treatment improved both the ICP and ICP_max_/MAP compared to the PBS group (Fig. 1A-B), demonstrating a similar effect to ADSC treatment. There was no significant difference in MAP among different groups. Furthermore, we tracked the location of hUC-MSC and ADSC using DIR probe or PKH26 stain. As shown in Fig. 1C, the transplanted hUC-MSC or ADSC gathered in the penis 24 h after transplantation; PKH26-labeled cells were observed in the penis 3 days after transplantation (Fig. 1D). Overall, our findings suggest that hUC-MSC transplantation effectively ameliorate erectile function in CNI-ED rats.

### hUC-MSC treatment promotes the expression of eNOS and nNOS in corpus cavernosum and increases the content of smooth muscle in penis

**Fig. 2:**
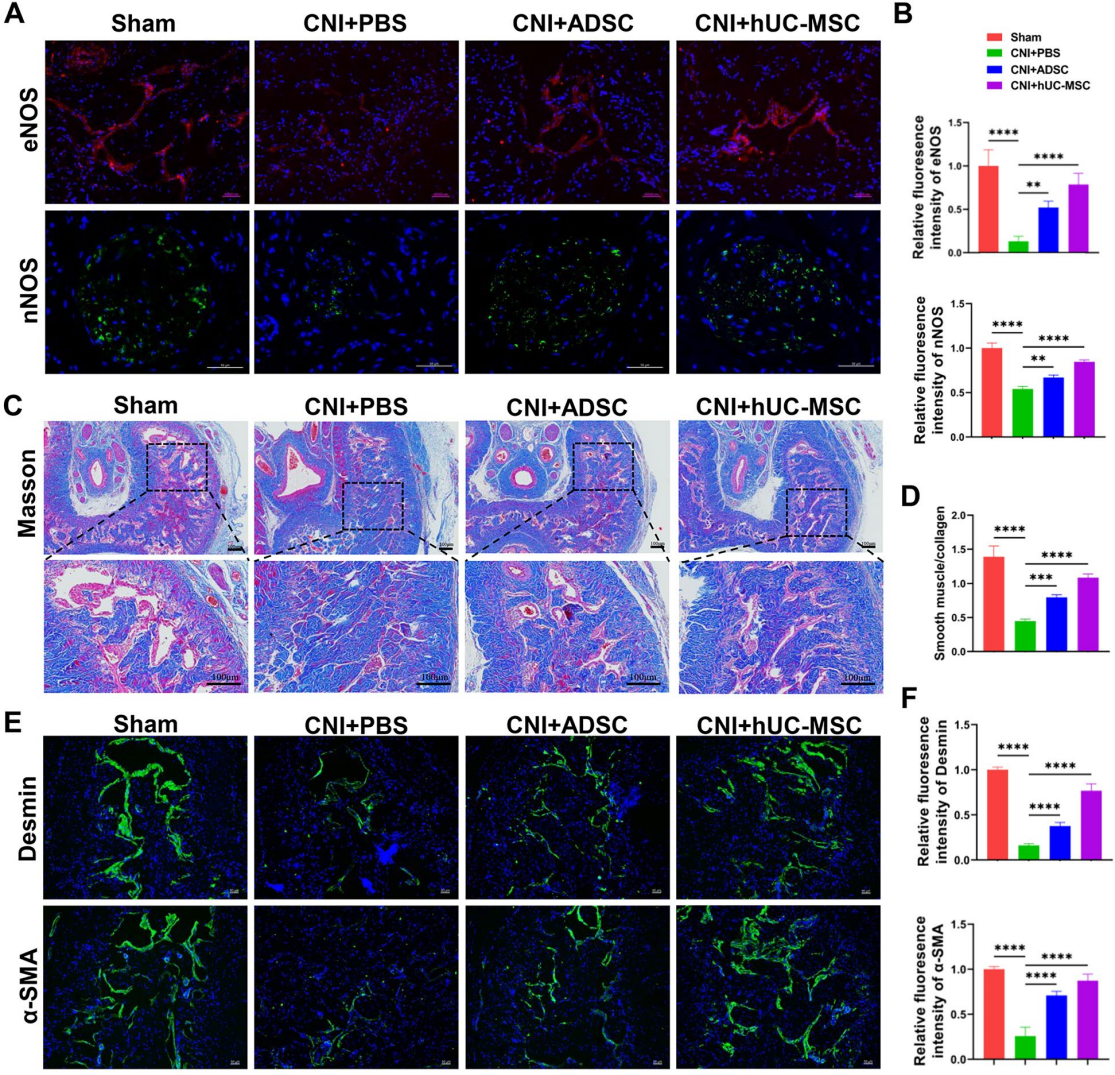
ADSC or hUC-MSC therapy improves the expression of eNOS and nNOS and increased the ingredient of smooth muscle in corpus cavernosum. **A** Immunostaining of corpora cavernosa with eNOS or nNOS antibody and the representative images are shown. Scale bar: 50 µm. **B** Quantification of immunofluorescence images of eNOS and nNOS from different groups. **C** Representative images of Masson trichrome staining of penile midshaft specimen in the four groups. The smooth muscle (red) and collagen (blue) tissues were stained by Masson Trichrome Kit. Scale bar: 100 µm. **D** Quantification of the ratio of smooth muscle to collagen. **E** Representative images of immunofluorescence of Desmin and α-SMA in different groups. Scale bar: 50 µm. **F** Quantification of immunofluorescence images of Desmin and α-SMA. The data of the above quantification of slides images are presented as mean ± SD, at least six representative visual fields in each slide for each group were counted. At least three rats were used in each group. (*p < 0.05, **p < 0.01, ***p < 0.001, ****p < 0.0001)

It’s well known that eNOS (a marker of endothelial function) and nNOS (a marker of cavernous nerve) play an important role in penile erectile physiology, which is closely linked to blood perfusion and penile erection [32]. The expression of eNOS and nNOS in corpus cavernosum was found to be decreased due to CNI. However, the administration of hUC-MSC or ADSC led to an increase in the expression of eNOS and nNOS (Fig. 2A-B).

The previous studies have demonstrated that CNI leads to a reduction in smooth muscle cell content [7, 33] and an increase in fibrosis of the corpus cavernosum [4]. To detect the content of smooth muscle cells and fibrosis degree in the corpus cavernosum, Masson staining was performed. As shown in Fig. 2C and D, there were more blue signals and fewer red signals observed in the corpus cavernosum of rats in CNI + PBS group compared to those in sham group. However, hUC-MSC treatment increased the content of smooth muscle cells and decreased the collagen deposition in the corpus cavernosum of rats (Fig. 2C, D). Consistent with the above observations, CNI decreased the expression of Desmin and α-SMA, two markers of smooth muscle cells; however, hUC-MSC treatment significantly increased their expression (Fig. 2E, F). Taken together, these data indicated that hUC-MSC administration could improve the function of endothelium and cavernous nerve, increase smooth muscle content, and reduces fibrosis of corpus cavernosum in CNI-ED rats.

### hUC-MSC treatment increases the mitochondrial mass of CCSMC in vivo and in vitro

**Fig. 3:**
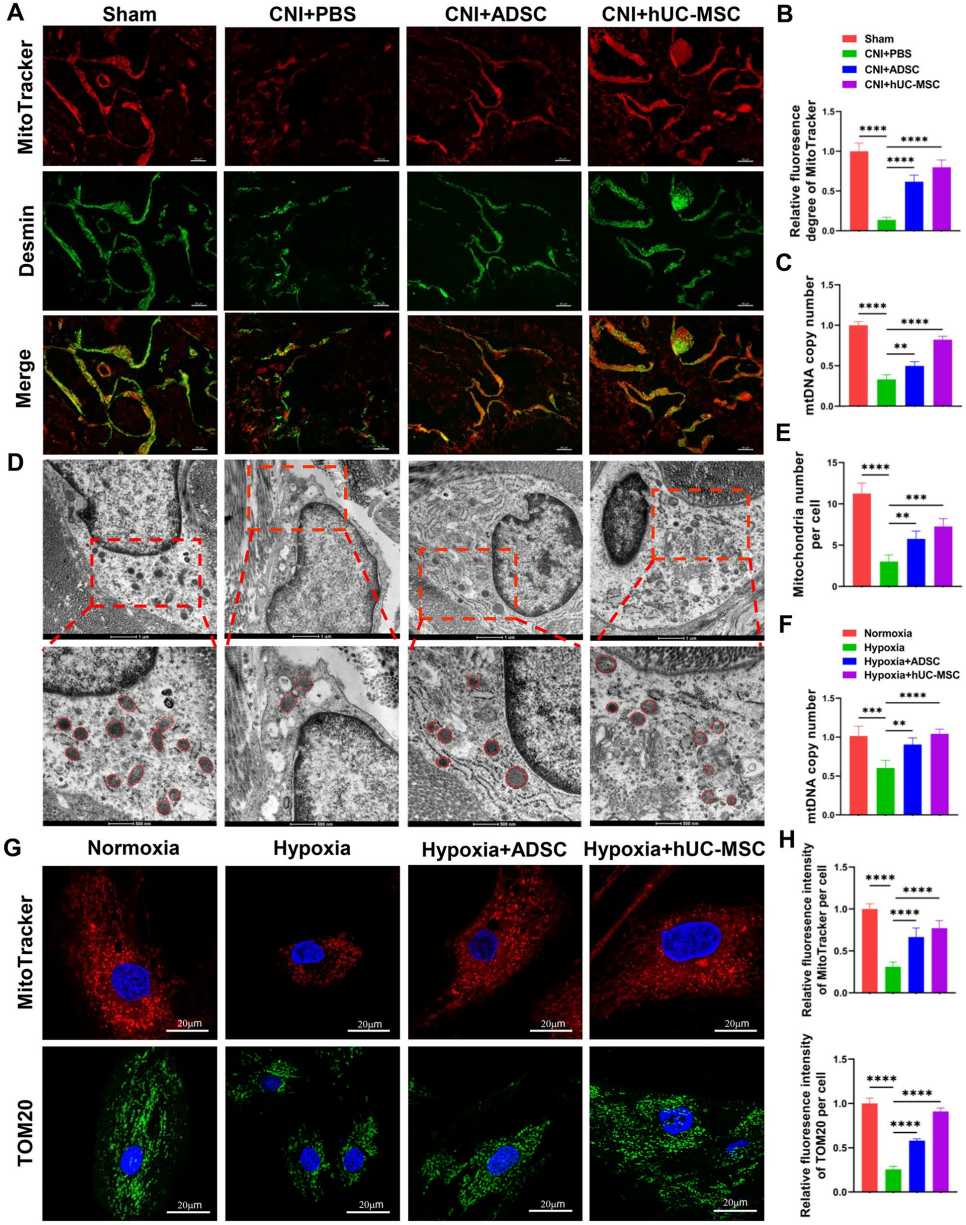
Treatment with ADSC or hUC-MSC improves mitochondrial mass and function in rat corpus cavernosum and in CCSMCs. **A** Representative images of immunofluorescence of penile cryosections stained using MitoTracker deep red (red) and Desmin (green) in CNI-ED. Scale bar: 50 µm. **B** Quantification of fluorescence density of the colocalization of MitoTracker deep red and Desmin. At least six representative visual fields were counted of each slide and at least three rats were used in each group. Data are presented as mean ± SD, **C** Real time analysis of mtDNA copy number in corpus cavernosum in different groups. mtDNA copy number in sham group were set as 1. At least 3 rats in each group were used, and the data are presented as mean ± SD. **D** Representative images of transmission electron microscopy (TEM) of mitochondria in CCSMCs. **E** Quantification of mitochondria number in different groups. At least 20 cells in each group were counted. **F** Quantification of mtDNA copy number in CCSMCs by real-time PCR. mtDNA copy number in normoxia group were set as 1. Data were collected from at least three independent experiments and the data are presented as mean ± SD. **G** Representative images of penile cryosections stained for MitoTracker deep red (red) and TOM20 (green) in CCSMCs. Scale bar: 20 µm. **H** Quantification of fluorescence density of MitoTracker deep red and in different groups. At least 20 cells were counted in each group and data are presented as mean ± SD. *p < 0.05, **p < 0.01, ***p < 0.001, ****p < 0.0001

Previous studies have demonstrated that erectile dysfunction is correlated with mitochondrial impairment [13, 14]. To investigate mitochondrial changes in the CNI-ED rat model, we examined the mitochondria in corpus cavernosum using MitoTracker Deep Red probe. Figure 3A and B shows that CNI led a reduction in mitochondrial mass in corpus cavernosum. However, treatment with ADSC or hUC-MSC increased the mitochondrial mass in corpus cavernosum, especially in CCSMCs (Fig. 3A-B). This phenomenon was further confirmed by assessing the mitochondrial DNA (mtDNA) copy number and the number of mitochondria per cell in corpus cavernosum by transmission electron microscopy (TEM) (Fig. 3C-E), however, treatment with ADSC or hUC-MSC can partially rescued this decrease (Fig. 3C-E). To further explore the beneficial effects of ADSC or hUC-MSC on the mitochondrial mass of CCSMCs, we initially cultured CCSMCs under hypoxia conditions (a sealed hypoxic jar) and subsequently co-cultured them with ADSC or hUC-MSC using a transwell system. As shown in Fig. 3F-H, hypoxia significantly reduced the mtDNA copy numbers and mitochondrial mass per cell of CCMSCs, as indicated by fluorescence intensity of MitoTracker Deep Red probe and TOM 20 expression levels. However, co-culture with ADSC or hUC-ADSCs increased mtDNA copy number and mitochondrial mass for CCSMCs. Taken together, these data suggested that hUC-MSC treatment could rescue the decrease of the mitochondrial mass in CCSMCs injured by CNI or hypoxia.

### hUC-MSC treatment improves mitochondrial function in corpus cavernosum and CCSMCs

**Fig. 4:**
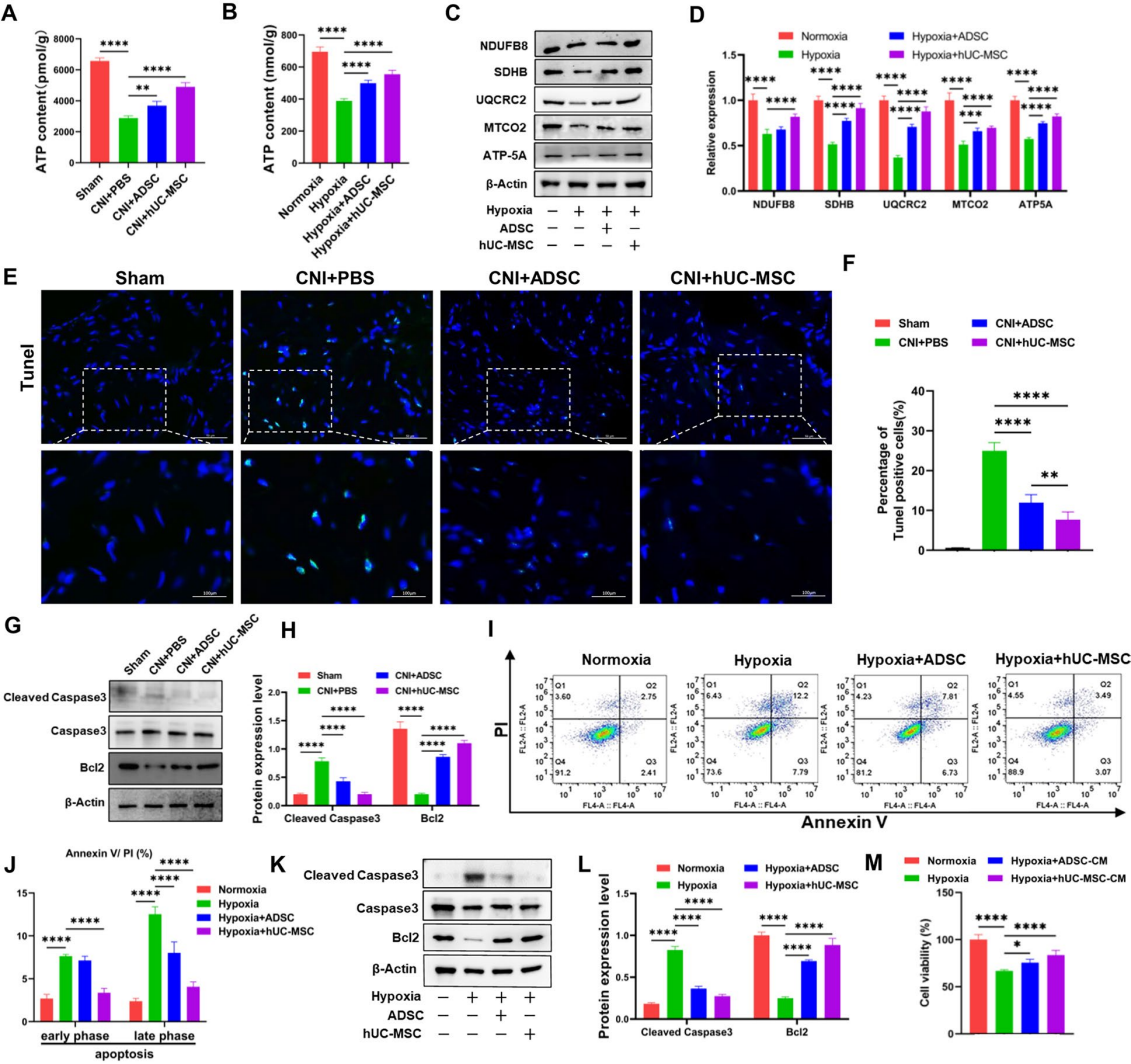
ADSC or hUC-MSC therapy alleviates mitochondrial dysfunction in corpus cavernosum and in CCSMCs. **A** ATP levels in corpus cavernosum of rats after ADSC or hUC-MSC injection. Data are presented as mean ± SD (n = 6). **B** Intracellular ATP levels in CCSMCs was measured after coculture with ADSC or hUC-MSC. Data were collected from at least three independent experiments, and data are presented as mean ± SD. **C** Immunoblot analysis of ETC protein levels including NDUFB8, SDHB, UQCRC2, MTCO2 and ATP5A in the lysates of CCSMCs. The level of b-actin was used as internal control of the total protein of cell lysate. Full-length blots of CCSMCs were presented (Additional file 2: Fig. S1). **D** Quantification of relative protein levels of NDUFB8, SDHB, UQCRC2, MTCO2 and ATP5A levels in CCSMCs. **E** Representative images of TUNEL staining of corpus cavernosum in different groups. Scale bar: 50 µm. **F** Quantification of TUNEL positive cells in all groups. At least six represent visual fields were counted for each group and at least 3 rats were used in each group. Data are presented as mean ± SD. **G** Immunoblot analysis of protein levels of Cleaved Caspase-3, Caspase-3 and Bcl-2 proteins in different groups. The level of b-actin was used as internal control of the total protein of cell lysate. Full-length blots of corpora cavernousa were presented (Additional file 2: Fig. S2). **H** Quantification of relative protein levels of Cleaved Caspase-3 and Bcl-2 levels in CCSMCs. **I** Flow cytometry analysis of Annexin V / PI staining of CCSMCs in different groups. **J** Quantification of apoptotic CCSMCs in different groups. Data were collected from at least three independent experiments and data are presented as mean ± SD. **K** Immunoblot analysis of protein levels of Cleaved caspase-3, Caspase-3 and Bcl-2 protein in CCSMCs. Full-length blots of CCSMCs were presented (Additional file 2: Fig. S3). **L** Quantification of relative protein levels of Cleaved caspase-3 and Bcl-2 levels in CCSMCs. **M** The cell viability of CCSMCs was assayed using Celltiter-Lumi™ Steady Luminescent Cell Viability Assay Kit. Data were collected from at least three independent experiments, and the data are presented as mean ± SD. *p < 0.05, **p < 0.01, ***p < 0.001, ****p < 0.0001

Mitochondria are organelles responsible for ATP production through oxidative phosphorylation and the regulation of intrinsic apoptosis [34]. To investigate changes in mitochondrial function of the corpus cavernosum, we measured ATP levels in the corpus cavernosum of CNI-ED rats. As shown in Fig. 4A, CNI significantly reduced ATP production in PBS group compared to that in sham group. However, treatment with ADSC or hUC-MSC evidently increased the ATP production in the corpus cavernosum. The conclusion was further confirmed in vitro using CCSMCs co-culture with ADSC or hUC-MSC under hypoxia conditions (Fig. 4B). Consistent with the ATP levels in CCSMCs, the expression levels of electronic transmission chain complex I-V proteins such as NDUFB8, SDHB, UQCRC2, MTCO2, and ATP5 were decreased when CCSMCs exposure to hypoxia. However, treatment with ADSC or hUC-MSC increased their expression (Fig. 4C and D).

Next, we further measured apoptosis in the corpus cavernosum. As shown in Fig. 4E, the number of TUNEL-positive cells in the corpus cavernosum were evidently higher in PBS group compared to Sham group. However, treatment with ADSC or hUC-MSC reduced the number of TUNEL-positive cells in corpus cavernosum (Fig. 4E and F). Additionally, CNI increased cleaved Caspase 3 expression while decreased Bcl2 expression in the corpus cavernosum, however hUC-MSC or ADSC treatment rescued their expression (Fig. 4G and H). Consistently, hUC-MSC or ADSC treatment also reversed hypoxia-induced early phase and late phase apoptosis of CCSMCs (Fig. 4I and J). Moreover, we confirmed these observations through assessment of the expression of cleaved Caspase3 and Bcl2 as well as cell viability (Fig. 4K-M). In summary, our data demonstrated that treatment with ADSC or hUC-MSC treatment improved mitochondrial function and reduced the apoptosis in both corpus cavernosum and CCSMCs induced by CNI or hypoxia.

### hUC-MSC treatment activates SIRT1/PGC-1α/TFAM pathway in CCSMCs

**Fig. 5:**
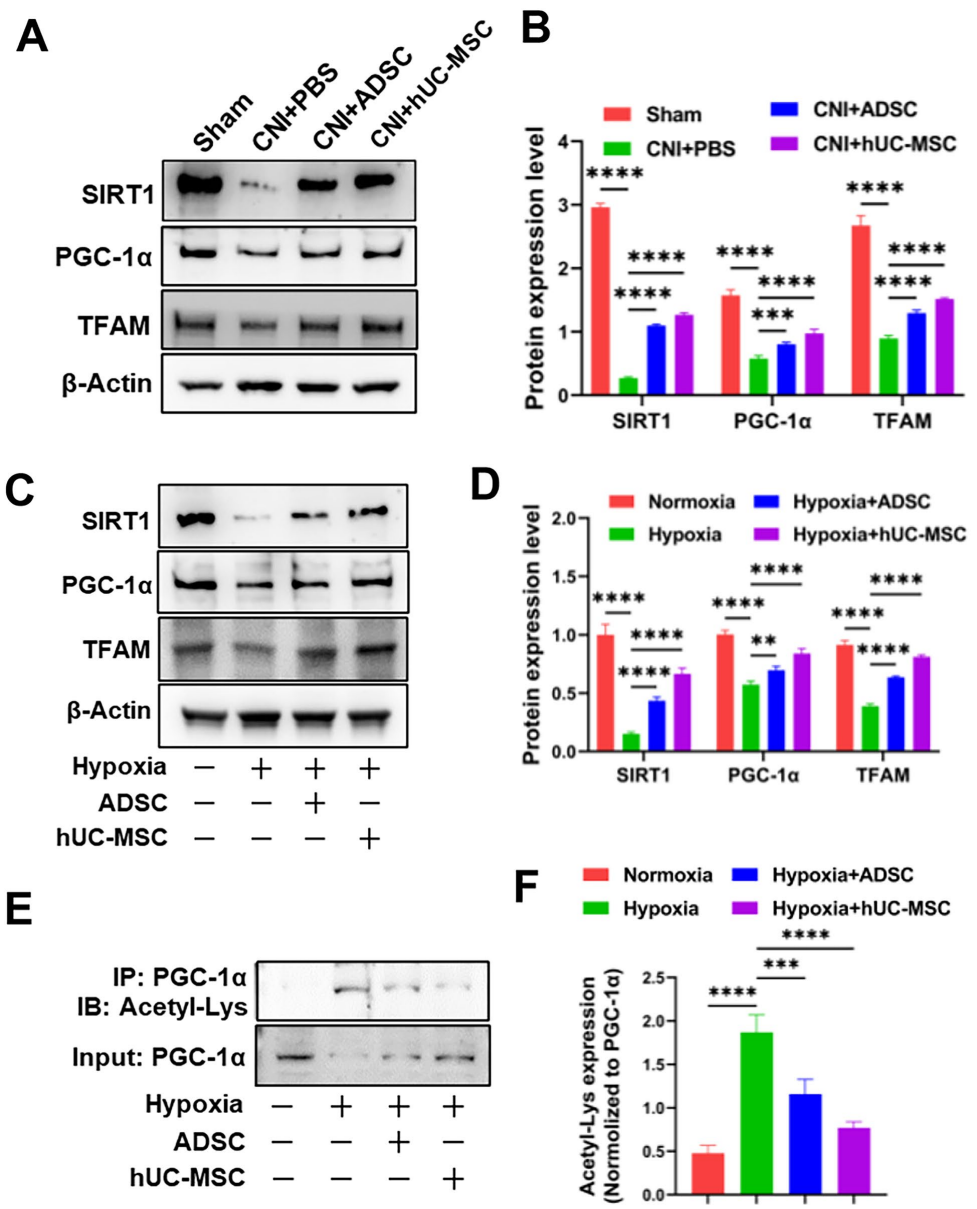
SIRT1/PGC-1α/TFAM pathway is upregulated after ADSC or hUC-MSC treatment. **A** Immunoblot analysis of protein levels of SIRT1, PGC-1α, TFAM in corpora cavernosa of rats. β-Actin expression levels were used as an internal control. Full-length blots of corpora cavernosa were presented (Additional file 2: Fig. S4). **B** Quantification of relative protein levels of SIRT1, PGC-1α, TFAM levels in corpora cavernosa. **C** Immunoblot analysis of protein levels of SIRT1, PGC-1α, TFAM expression levels in CCSMCs. β-Actin expression levels were used as an internal control. Full-length blots of CCSMCs were presented (Additional file 2: Fig. S5). **D** Quantification of relative protein levels of SIRT1, PGC-1α, TFAM levels in CCSMCs. **E** Immunoprecipitation analysis of acetylation level of PGC-1α in CCSMCs. Full-length blots of CCSMCs were presented (Additional file 2: Fig. S6). **F** Quantification of acetylation level of PGC-1α in CCSMCs. Acetylated level of PGC-1α was normalized to PGC-1α. *p < 0.05, **p < 0.01, ***p < 0.001, ****p < 0.0001)

SIRT1/PGC-1α/TFAM pathway have been shown to play a critical role in regulating mitochondrial biogenesis [29, 30], therefore, we evaluated the SIRT1/PGC-1α/TFAM pathway in CNI-ED rats. As shown in Fig. 5A and B, the expression levels of SIRT1, PGC-1α, and TFAM of corpus cavernosum were significantly lower in the PBS group than those in the sham group. However, administration of ADSC or hUC-MSC increased their expression. This finding was further confirmed by an in vitro experiment using CCSMCs co-cultured with ADSC or hUC-MSC exposed to hypoxia (Fig. 5C and D). Furthermore, previous studies have demonstrated that SIRT1 mediates the deacetylation of PGC-1α, leading to its activation and subsequently promotion of TFAM transcription [29, 35]. To investigate the mechanism, we performed immunoprecipitation experiments using PGC-1α antibody on CCSMCs lysates in vitro and assessed the acetylation level of PGC-1α with acetyl-lysine. Figure 5E and F shows that hypoxia increased the acetylation level of PGC-1α; however, treatment with ADSC or hUC-MSC decreased the acetylation level of PGC-1α. Taken together, these data suggest that administration of ADSC or hUC-MSC can reverse the downregulation of the SIRT1/PGC-1α/TFAM pathway induced by CNI and hypoxia, leading to an increase in mitochondrial biogenesis and mitochondrial mass within CCSMCs.

### SIRT1 knockdown abrogates the protective effects of hUC-MSC on the mitochondrial mass of CCSMCs

**Fig. 6:**
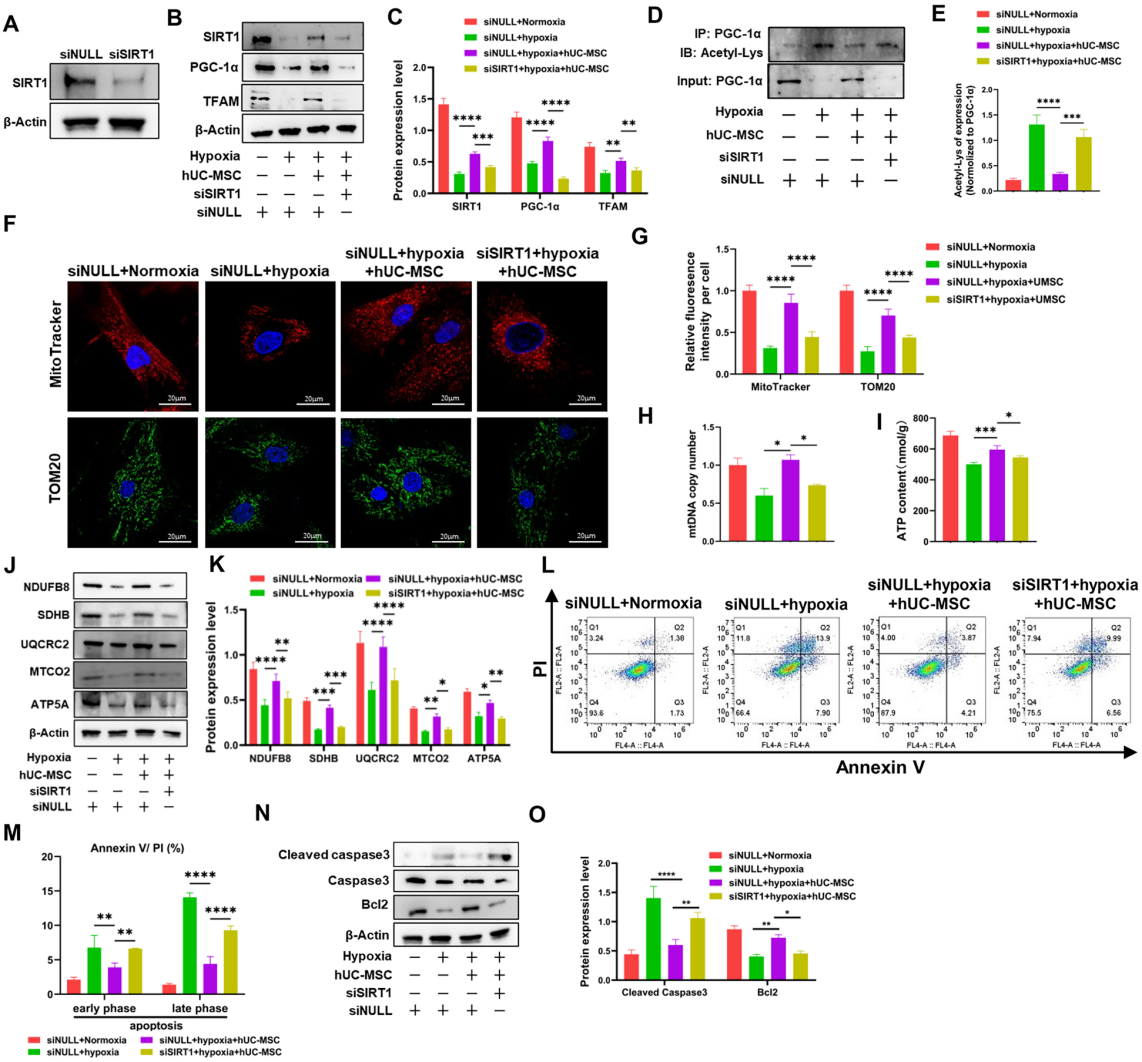
MSC restore mitochondrial biogenesis and function via SIRT1/PGC-1a/TFAM pathway. **A** Immunoblot analysis of protein level of SIRT1 expression in CCSMCs after SIRT1 knockdown by siRNA. β-Actin expression levels were used as an internal control. Full-length blots of CCSMCs were presented (Additional file 2: Fig. S7). **B** Immunoblot analysis of protein levels of SIRT1, PGC-1α, TFAM in CCSMCs of different groups. Full-length blots of CCSMCs were presented (Additional file 2: Fig. S8). **C** Quantification of relative protein levels of SIRT1, PGC-1α, TFAM levels in different groups. **D** Immunoprecipitation analysis of acetylation level of PGC-1α in CCSMCs. Full-length blots of CCSMCs were presented (Additional file 2: Fig. S8). **E** Quantification of acetylation level of PGC-1α in different groups. Acetylation level of PGC-1α were normalized to PGC-1α. **F** Representative images of mitochondria of CCSMCs stained with MitoTracker deep red (red) or anti-TOM20 monoclonal antibody (green). Scale bar: 20 µm. **G** Quantification of relative fluorescence intensity of MitoTracker deep red or TOM20 of per cell in CCSMCs in different groups. At least 20 cells were counted for each group and data are presented as mean ± SD. **H** Quantification of the mtDNA copy number in CCSMCs. Data were collected from at least three independent experiments, and the data are presented as mean ± SD. **I** Intracellular ATP level in CCSMCs after knockdown of SIRT1 and coculture with hUC-MSCs. Data were collected from at least three independent experiments and data are presented as mean ± SD. **J** Immunoblot analysis of protein levels of NDUFB8, SDHB, UQCRC2, MTCO2 and ATP5A in CCSMCs in different groups. Full-length blots of CCSMCs were presented (Additional file 2: Fig. S10). **K** Quantification of relative protein levels of NDUFB8, SDHB, UQCRC2, MTCO2 and ATP5A levels of CCSMCs in different groups. **L** Flow cytometry analysis of Annexin V / PI staining of CCSMCs in different groups. **M** Quantification of apoptotic CCSMCs in different groups. Data were collected from at least independent groups, and the data are presented as mean ± SD. **N** Immunoblot analysis of protein levels of Cleaved caspase-3, Caspase-3 and Bcl-2 protein in CCSMCs. Full-length blots were presented (Additional file 2: Fig. S11). **O** Quantification of relative protein levels of Cleaved caspase-3 and Bcl-2 levels in CCSMCs. *p < 0.05, **p < 0.01, ***p < 0.001, ****p < 0.0001

To investigate the potential role of SIRT1/PGC-1α/TFAM pathway in mediating hUC-MSC regulation of mitochondrial mass in CCSMCs, we utilized siRNA to knock down SIRT1 in CCSMCs, with siNULL serving as a control. The protein level of SIRT1 was significantly reduced in SIRT1 siRNA (siSIRT1) group compared to the siNULL group (Fig. 6A). Subsequently, under hypoxia condition, we observed a marked decrease in the expression of SIRT1, PGC-1a and TFAM. However, co-culture with hUC-MSC increased their expression. Notably, knockdown of SIRT1 reversed the increase in this protein expression (Fig. 6B, C). Consistently, knockdown of SIRT1 enhanced acetylation of PGC-1a in hypoxia-exposed CCSMCs when treated with hUC-MSC (Fig. 6D, E). Furthermore, we further observed the mitochondrial mass in CCSMCs and confirmed that knockdown of SIRT1 eliminated the increase seen in mitochondrial mass within hypoxia-exposed CCSMCs when treated with hUC-MSC. This was evidenced by a decreased fluorescence intensity of MitoTracker Deep Red probe and TOM 20 expression (Fig. 6F, G), as well as a similar trend for mtDNA copy number (Fig. 6H). We also assessed the mitochondrial function of CCSMCs through ATP production (Fig. 6I), ETC complex proteins (Fig. 6J, K), and apoptosis (Fig. 6L-O), all yielding consistent results. Overall, the data indicates that the beneficial of hUC-MSC on the mitochondrial mass and function within hypoxia-injured CCSMCs may be mediated by SIRT1/PGC-1α/TFAM pathway.

## Discussion

CNI-ED is a distressing complication that severely undermines the psychological and physical well-being of patients [36]. As the primary structural and functional unit for sustaining penile erection, a reduction in CCSMCs results in the failure of the remaining corporal smooth muscle mass to relax adequately [37]. Increased apoptosis of CCSMCs is a significant factor contributing to the development of CNI-ED. In the present study, we first demonstrated that hUC-MSC treatment can enhance erection function in the CNI-ED rats by restoring the content of CCSMCs and reduced their apoptosis. Furthermore, the therapeutical effect of hUC-MSC is comparable to that of ADSC. It is important that hUC-MSC are readily accessible and do not require any invasive surgical procedures. Consequently, hUC-MSC represent a promising resource for cell therapy in the clinical treatment of CNI-ED in the future.

Increasing evidence has demonstrated that mitochondrial impairment plays an important role in the development of in various diseases [38, 39]. Our study revealed that Desmin-positive cells exhibited higher mitochondrial mass compared to other cell types within the corpus cavernosum. Furthermore, a notable decrease in mitochondrial mass was observed in Desmin positive cells from corpus cavernosum of CNI-ED rats, indicating the reduction of mitochondrial mass in CCSMCs is a primary characteristic of the CNI-ED rat model. In vitro experiments demonstrate that hypoxia significantly reduces both mitochondrial mass and function of CCSMCs, which is accompanied by a notable increase in apoptosis. Conversely, hUC-MSC enhance the mitochondrial mass and function, while mitigating the apoptosis of CCSMC, both in vivo and in vitro. This results in increased smooth muscle content as well as a higher ratio of smooth muscle to collagen. Supporting our study, recent studies have reported that ameliorating mitochondrial dysfunction and apoptosis in CCSMCs through Caveolin-1 scaffolding domain (CSD)-derived peptide alleviates CNI-ED [37].

SIRT1/PGC-1a/TFAM signaling pathway has been well-documented as a crucial regulator of mitochondrial biogenesis and bioenergetics [19, 27, 28, 40]. Our study confirms that both CNI and hypoxia lead to the downregulation of SIRT1, PGC-1a and TFAM expression. This results in a decrease in mitochondrial mass and function, ultimately promoting cell apoptosis. These finding are consistent with previous studies indicating that hypoxia reduces SIRT1 expression, which contributes to mitochondrial dysfunction, cellular apoptosis, and fibrosis [41, 42]. Furthermore, the inhibition of SIRT1 expression in CCSMCs has been associated with erectile dysfunction [43]. Our findings demonstrate that hUC-MSC upregulates the expression of SIRT1, PGC-1a, and TFAM. Notably, the knockdown of SIRT1 in CCSMCs abolishes the activating effect of hUC-MSC on the SIRT1/PGC-1α/TFAM signaling pathway and diminishes its protective effect on the mitochondrial mass and function of CCSMCs subjected to hypoxia Therefore, hUC-MSC enhances mitochondrial mass and restores mitochondrial function by activating SIRT1/PGC-1α/TFAM pathway. However, silencing SIRT1 does not completely reverse the effects of hUC-MSC on mitochondrial mass and function, suggesting that additional pathway may be involved in this process. Consequently, further research is required to explore the underlying molecular mechanism at play.

In the present study, we employed a transwell system to culture hUC-MSCs. Consequently, we hypothesized that the secretions from the cells would upregulate SIRT1/PGC-1α/TFAM signaling pathway. This hypothesis is supported by previous research demonstrating that the secretions of MSCs can enhance mitochondrial function through the activation of SIRT1/PGC-1α pathway, which results in reduced cell apoptosis and restoration of cellular function [27, 44]. Furthermore, additional studies have indicated that the secretions from MSCs activate the phosphorylation of PI3K and Akt, leading to a direct interaction between Akt and SIRT1, thereby promoting an increase in SIRT1 expression [27]. However, further investigation is required to identify the specific factors within hUC-MSC secretions responsible for upregulating SIRT1 expression. In addition, further signaling pathways involved in the mitochondrial biogenesis can be investigated through profiling analysis. Unfortunately, human CCSMCs were not utilized for in vitro experiments due to challenges in acquisition. There is an urgent necessity to investigate the clinical therapeutic effects of hUC-MSCs on CNI-ED, and we are eager to expedite the process of clinical translation as soon as possible.

## Conclusions

**Fig. 7:**
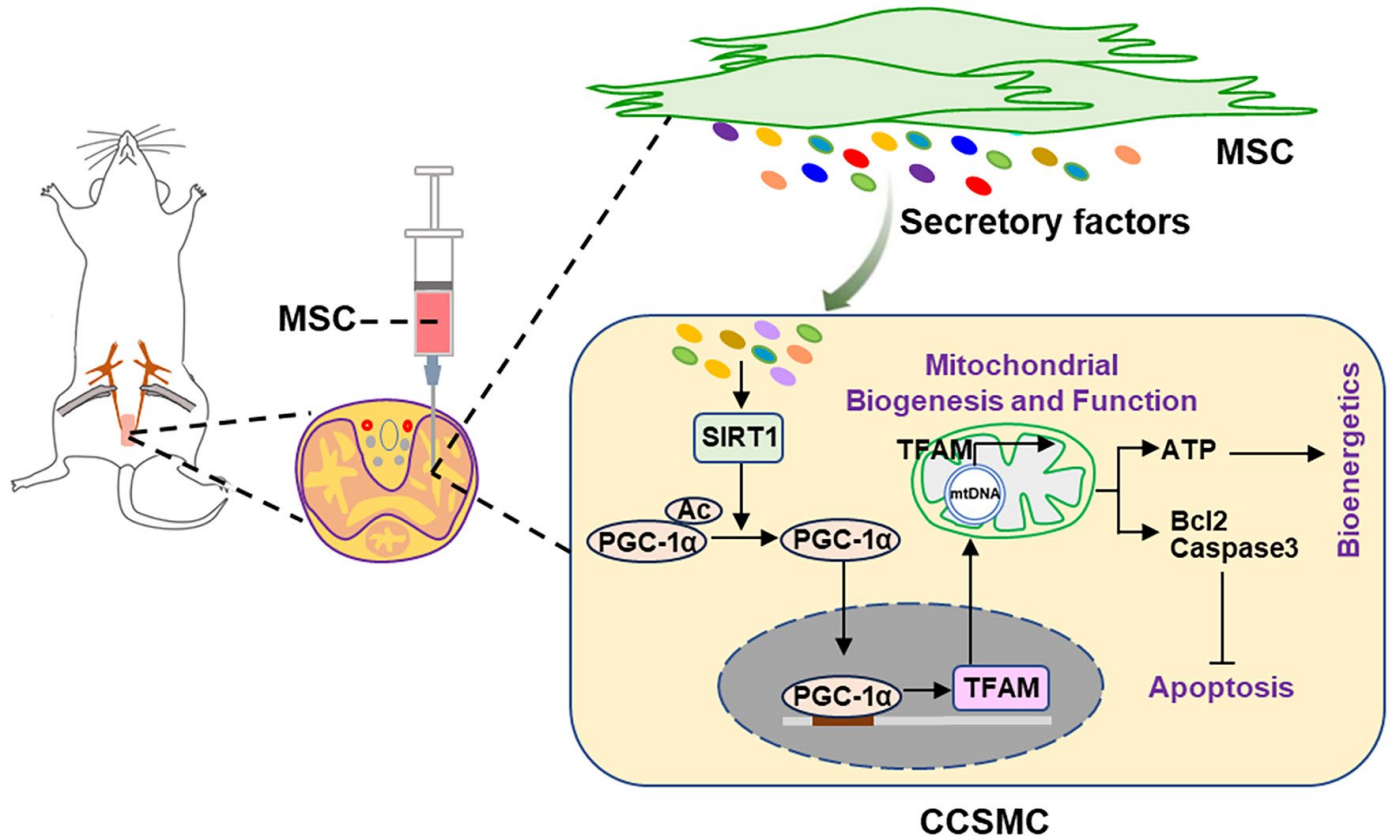
Working model of MSC in alleviating CNI-ED rats. Rats are subjected to bilateral cavernous nerve crush and followed by intracavernous injection of MSC. The secretions from MSC activate SIRT1 expression, which subsequently deacetylates acetylated PGC-1α and activates PGC-1α. The activated PGC-1α then translocated into the nuclei, where it promotes the transcription of TFAM. TFAM plays a crucial role in mitochondrial biogenesis and maintaining mitochondrial homeostasis, thereby enhancing mitochondrial bioenergetics and reducing cell apoptosis. Ultimately, this process contributes to the restoration of erectile function

Our study has demonstrated that hUC-MSC improve erectile function in CNI-ED rats and enhance both mitochondrial mass and function of CCSMCs. Mechanistically, the SIRT1/PGC-1a/TFAM pathway mediates the beneficial effects of hUC-MSC on CCSMCs through secreting certain factors or cytokines from hUC-MSC (Fig. 7). Therefore, our findings provide new insights into understanding the role and molecular mechanisms of MSCs in restoring erectile function in CNI-ED. Furthermore, targeting mitochondrial dysfunction may represent a promising therapeutic strategy for alleviating CNI-ED.

## Materials and methods

### Isolation and culture of hUC-MSC and ADSC

The hUC-MSC were isolated as reported previously [45] and cultured in α-MEM medium containing of 10% FBS and 1% P/S. The women gave informed consent for the sample harvest. ADSCs were isolated as reported previously [46–48] and cultured in DMEM/F12 medium containing 10% fetal bovine serum (FBS); and 1% penicillin/streptomycin (P/S) (all from Thermo Fisher). The collection and subsequent use of fatty tissues or umbilical cord tissues was approved by the Human and Animal Research Ethic Commitment of Renji Hospital, School of Medicine, Shanghai Jiao Tong University.

### Immunophenotype analysis of hUC-MSC and ADSC

hUC-MSC and ADSC were digested and washed with cold PBS twice, 1 × 10^6^ cells were stained with the following fluorescein-conjugated antibodies: CD105-APC, CD90-FITC, CD73-FITC, CD34-PerCP, CD31-FITC, HLA-DR-FITC (all form BD Bioscience) for 30 min at 4 ^◦^C. Then, the cells washed with PBS and resuspended in PBS for flow cytometric analysis. At least 10^5^ events were acquired using BD Accuri^™^ C6 Flow cytometer (BD Bioscience).

### Osteogenic and adipogenic differentiation of hUC-MSC and ADSC

To assess the adipogenic and osteogenic differentiation potential, cells at 90% confluency were changed with osteogenic or adipogenic differentiation medium (all from STEMCELL Technologies), and then were identified by alizarin red staining and Oil Red-O staining, respectively.

### hUC-MSC or ADSC treatment of CNI-ED rat model

Eight-week-old male SD rats weighting approximately 240–260 g were utilized to establish CNI-ED model according to previous report [31, 49]. The rats were purchased from Shanghai Jihui Co., Ltd and housed under specific pathogen-free conditions with 12 h light and dark cycle and free access to food and water. All animal experiments were approved by the Human and Animal Research Ethics Commitment of Renji Hospital affiliated to Shanghai Jiao Tong University School of Medicine (NO: KY2022-180-B).

All rats were randomly divided into four groups (n = 6): sham, CNI + PBS, CNI + ADSC, and CNI + hUC-MSC. The CNI-ED model was established according to previous report [31]. Briefly, SD rats were anaesthetized with 40 mg/ kg pentobarbital sodium and the rats in sham group were performed a lower abdominal midline incision and suture. The rats in CNI + PBS, CNI + ADSC, and CNI + hUC-MSC group were subjected to a lower abdominal midline incision, and then received a crush on bilateral cavernous nerves (3 mm distal to the major pelvic ganglion) for 120 s by micro forceps. Then, the rats in PBS group received intracavernous injection of PBS (0.1 ml), and the rats in CNI + ADSC group or CNI + hUC-MSC group received ADSC and hUC-MSC extracavernous injection (1 × 10^6^ cells in PBS 0.1 ml), respectively. Before injection, the penis base was tethered with rubber tourniquet, and 1 min after injection, the tourniquet was released. After injection, abdominal incisions were sterilized and sutured. 4 weeks later, the erectile function of rats in different groups was evaluated by electrical stimulation device.

### Erectile function assessment

To evaluate erectile function of the rats, the rats were anaesthetized, and the left carotid artery were isolated. Then, a heparinized 24-gauge silastic cannula connected to the left carotid artery and a pressure transducer was used to measure the mean arterial pressure (MAP). Then, the major pelvic ganglion and cavernous nerve were carefully isolated from the prostate with the bipolar hook electrode. Subsequently, the muscle in bulbospongiosus and ischiocavernosus were stripped away using a curved forceps, to exposing the white tunica albuginea of the crus of the corpus cavernosum. Lastly, a heparinized needle was connected to a catheter and a pressure transducer and inserted into the crus corpus cavernosum through the white tunica albuginea to measure intracavernosal pressure (ICP). The cavernous nerve was stimulated (5 V and 15 Hz for 1 min) with a bipolar hook electrode, and the changes of MAP and ICP were recorded using BL-420 biological function system (Chengdu Taimeng Technology Co., Ltd.). The interval between stimulations was at least 15 min. The maximal intracavernosal pressure (ICP_max_)/ MAP was used to measure erectile function.

### Histology and immunofluorescence staining analysis

For masson’s trichrome staining, the penile mid-shaft tissues were fixed with 4% paraformaldehyde (PFA; Beyotime technology, China) for 24 h and embedded in paraffin. Subsequently they were cut into sections with a thickness of 5 μm. The sections were deparaffinized in xylene and hydration with gradient alcohol. They were then stained using masson’s trichrome staining kit (Solarbio, China) following the manufacturer’s instructions. Briefly, the sections were stained with Weigert iron hematoxylin solution for 2 min (min) and washed for 3 min. Next, the sections were stained with Ponceau-acid fuchsin solution for 5 min, followed by differentiation with phosphomolybdic acid solution for 2 min. They were then incubated with aniline blue solution staining for 2 min. After that, the sections were incubated for 3 min in 1% glacial acetic acid to enhance color delicacy and transparency. The sections were then washed with distilled water, dehydrated with gradient alcohol, and cleared before being mounted in dibutyl phthalate xylene. The corpus cavernosum smooth muscle cells were stained into red while the collagen fibers were stained into blue. The stained images were visualized with an inverted microscope.

For immunofluorescence staining, the mid-shaft penile tissues were harvested and fixed with 4% PFA for 24 h. Subsequently, they were dehydrated in a 30% sucrose/PBS solution and then embedded in OCT (SAKURA). The tissues were cut into sections with a thickness of 10 μm. The frozen slides were incubated with PBS for 10 min to remove OCT, and permeated with 0.3% Triton X-100 at RT for 15 min, and then blocked with donkey serum for 30 min at RT. Next, they were incubated at 4^◦^C overnight with the primary antibodies as follows: anti-nNOS, anti-eNOS, anti-desmin, anti-TOM20 (all from Abcam), anti-α-SMA (Cell Signaling Technology). Next, the sections were washed with PBS three times and incubated with corresponding 488- or 594-coupled secondary antibody (abcam) for 1 h at RT and washed with PBS for 3 times. The images were visualized using an inverted fluorescence microscope, and Image J software was used to quantify the density of immunostaining on the slides.

For mitochondrial staining, the sections were incubated with 100 nM MitoTracker deep red probe (Thermo Fisher) for 30 min at RT as previously reported [50], and washed three times with PBS, then stained cell nuclei with 2 μg/mL DAPI (Sigma) for 5 min at RT. The slides were then visualized using confocal fluorescence microscope (Olympus FV3000). For quantification analysis, the areas of the slide were randomly selected, and staining were repeated using at least 10 representative sections from at least three rats for each group, and the representative images were shown in the figures, and the statistical analysis was performed by two readers who were blinded to the slide. Image J was used for image analysis.

### Isolation and culture of CCSMCs

CCSMCs were isolated according to the previous report [51]. Briefly, the rats were anaesthetized, and their penis were cut open. After removal of urethra, glans, dorsal blood vessels and nerves, the corpus cavernosum tissues were washed with cold PBS and cut into 1–2 mm pieces. The segments were seeded into the six-well plate, supplemented with a minimal volume of DMEM medium (GIBCO) containing 20% FBS and cultured in a cell incubator (Thermo Fisher) in 5% CO2 at 37 °C. After the explants adhered to the bottom, more DMEM culture medium containing10% FBS was added. The CCSMCs were passaged or frozened when achieved 80%−90% confluence.

### Collection the conditioned medium (CM) of hUC-MSCs and ADSCs

hUC-MSCs or ADSCs were cultured to a density at 80% and changed the medium with fresh serum-free α-MEM medium and cultured for another 24 h, then collected the supernatant. The supernatant was centrifugated at 2000 rpm/10 min to remove cell debris and was used for the subsequent experiments.

### Cell viability

To test cell viability, hUC-MSC-CM and ADSC-CM was concentrated using a 10 kDa ultracentrifugation centrifugal filter (Merck Millipore). Next, CCSMCs (2000/well) were seeded in a 96-well plate and pretreated with 50% hUC-MSC-CM or ADSC-CM and 50% DMEM with 10% FBS for 2 h, then CCSMCs were placed in a sealed hypoxic jar at 37 ^◦^C for 24 h. Subsequently, the cell viability was tested by CellTiter-Lumi^™^ Steady Luminescent Cell Viability Assay Kit (Beyotime Biotechnology) according to the manufacturer’s instruction.

### Small interfering RNA (siRNA)

For RNA silencing, the sequence of siRNA targeting rat SIRT1 was as follows: 5’-AAGTGCCTCAAATATTAATAA-3’. A negative control siRNA (siNULL) sequence was used as follows: 5’-UUCUGGGAACACGAGUGCU-3’. CCSMCs at 50% confluence were utilized for transfection with 50 nM siSIRT1 RNA or siNULL using TSnanofect V1 transfection Reagent following the manufacturer’s manual.

### Measurement of mtDNA copy number

For relative mtDNA copy number detection, total DNA extractions were prepared from corpus cavernosum or CCSMCs TIANamp Genomic DNA Kit (TianGen, China). Quantification of mtDNA levels was performed using SYBR-Green qPCR analysis and primers targeting tRNA and GAPDH as previously described [52].

### Immunoprecipitation

The immunoprecipitation assay was performed using Pierce magnetic IP/co-IP kit (Thermo fisher). CCSMCs were lysed with RIPA on the ice for 30 min. The protein quantification was conducted with a BCA protein quantification kit (Thermo fisher). Equal amounts of protein lysates (300 μg) were incubated with 10 μg PGC-1α antibody (Novus Biologicals) with rotation overnight at 4 ℃ to immunoprecipitate PGC-1α protein. Next, the cell lysates were added to pre-washed protein A/G magnetic beads slurry and mixed with rotation for 30 min at 4 ℃ to capture PGC-1α antibody. Then, the cell lysates were placed into a magnetic stand to collect the beads, and then washed twice. Finally, the beads were eluted by low-pH elution buffer and collected for immunoblot with acetylated-Lysine antibody (CST).

### Western blot analysis

The tissues or CCSMCs were lysed with RIPA (Beyotime Biotechnology, China) at 4 ^◦^C for 30 min, then centrifugated at 12000 rpm/10 min at 4 ◦C to harvest the supernatant. The total protein concentration was measured by BCA protein assay kit. The proteins were denatured by loading buffer at 100 ℃for 5 min and separated using SDS-PAGE gel and then transferred to the PVDF membrane (Millipore). Next, the PVDF membrane was blocked with 5% non-fat milk for 1 h at RT and incubated with primary antibodies at 4 ℃ overnight. The information of the primary antibodies is as follows: SIRT1 (Abcam), TOM20 (Abcam), PGC-1a (Novus Biologicals), MTCO2, UQCRC2, SDHB, NDUFB8, ATP5A, β-Actin (above all from Proteintech), Cleaved caspase3, Caspase3, Bcl2 (above all from Abclonal). After washing with TBST three times, corresponding HRP-conjugated secondary antibody (Proteintech) was incubated for 1 h at RT, then washing three times and the bands were visualized through an enhanced chemiluminescence substrate (Millipore). The information of primary antibodies was listed in Table S1.

### Statistical analysis

All quantified data were presented as mean ± SD of at least three independent experiments. The statistical analysis was assessed by SPSS software 22.0, and statistical significance was determined using Student’s t-tests for comparison of two groups and using one-way ANOVA with Tukey’s post hoc test for three and more groups comparisons. The data that followed a normal distribution were analyzed using parametric methods, while the Kruskal–Wallis non-parametric test was employed for data that did not conform to a normal distribution. Statistically significance was set as. *P < 0.05, **P < 0.01, ***P < 0.001, and ****P < 0.0001.

## Supplementary Information

Additional file 1

Additional file 2

## Data Availability

The data for this study are available within the article, with additional data available in the Supporting Information, or are available from the corresponding author on reasonable request.

## Abbreviations

ED: Erectile dysfunction
MSC: Mesenchymal stem cells
CN: Cavernous nerves
CNI: Cavernous nerve injury
CNI-ED: Cavernous nerve injury-induced erectile dysfunction
CCSMC: Corpus cavernous smooth muscle cells
RP: Radical prostatectomy
MSC: Mesenchymal stem cell
hUC-MSC: Human umbilical cord mesenchymal stem cell
ADSC: Adipose-derived stem cells
CM: Conditioned medium
MSCCM: Mesenchymal stem cell-conditioned medium
hUC-MSC-CM: Human umbilical cord mesenchymal stem cell-conditioned medium
ADSC-CM: Adipose-derived stem cells-conditioned medium
MAP: Mean arterial pressure
ICP: Intracavernosal pressure
ICPmax: Maximal intracavernosal pressure
siRNA: Small interfering RNA
