# Supplementary material for: hUC-MSC preserves erectile function by restoring mitochondrial mass of penile smooth muscle cells in a rat model of cavernous nerve injury via SIRT1/PGC-1a/TFAM signaling
Source: Biol Res. 2025 Jan 27;58:8. doi: 10.1186/s40659-024-00578-y (PMC11773750; doi:10.1186/s40659-024-00578-y)
Supplement: Supplementary file 1 — Additional file 1 [file 40659_2024_578_MOESM1_ESM.docx]

**hUC-MSC preserves erectile function by restoring mitochondrial mass of penile smooth muscle cells in a rat model of cavernous nerve injury via SIRT1/PGC-1α/TFAM signaling**

Mengbo Yang^1^^#^, Xinda Chen^1#^, Ming Zhang^1#^, Xiaolin Zhang^1^, Dongdong Xiao^1*^, Huiming Xu^2*^, Mujun Lu^1^^*^

Supplemental Information

## Supplemental Figures and Figure Legends


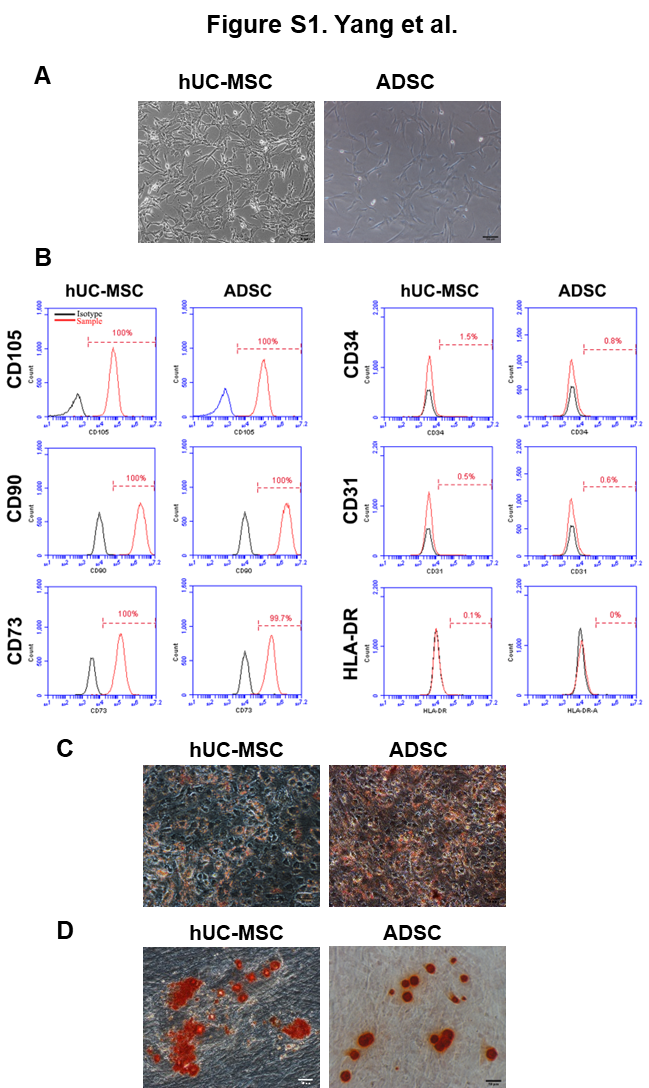


Figure S1. Characterization of hUC-MSCs and ADSCs and differentiation potential. (A) Morphology of hUC-MSCs and ADSCs. (B) Characterization of UC-MSCs and ADSC by flow cytometry with antibodies against CD105, CD90, CD73, CD45, CD31 and HLA-DR. (C) hUC-MSC or ADSC were differentiated into adipocytes for 21 days and then analyzed oil Red-O staining. Scale bar, 50 μm. (D) hUC-MSC or ADSC were differentiated into osteoblasts for 15 days and then analyzed by alizarin red staining. scale bar, 50 µm.


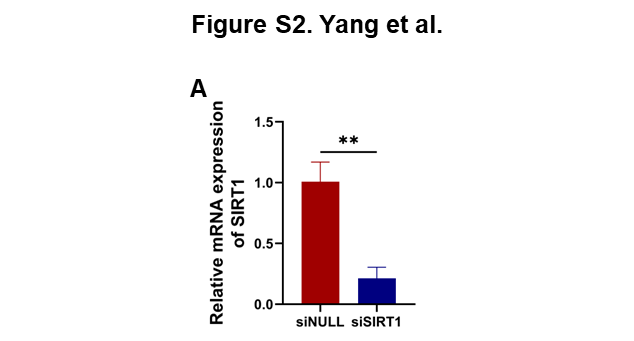


Figure S2. mRNA levels of SIRT1 after SIRT1 siRNA treatment. CCSMCs were transfected with 100uM siRNA of SIRT1 for 24h, and qualification of relative mRNA level of SIRT1 in the CCSMCs. The data were collected from at least three independent experiments and the data are presented as mean ± SD.


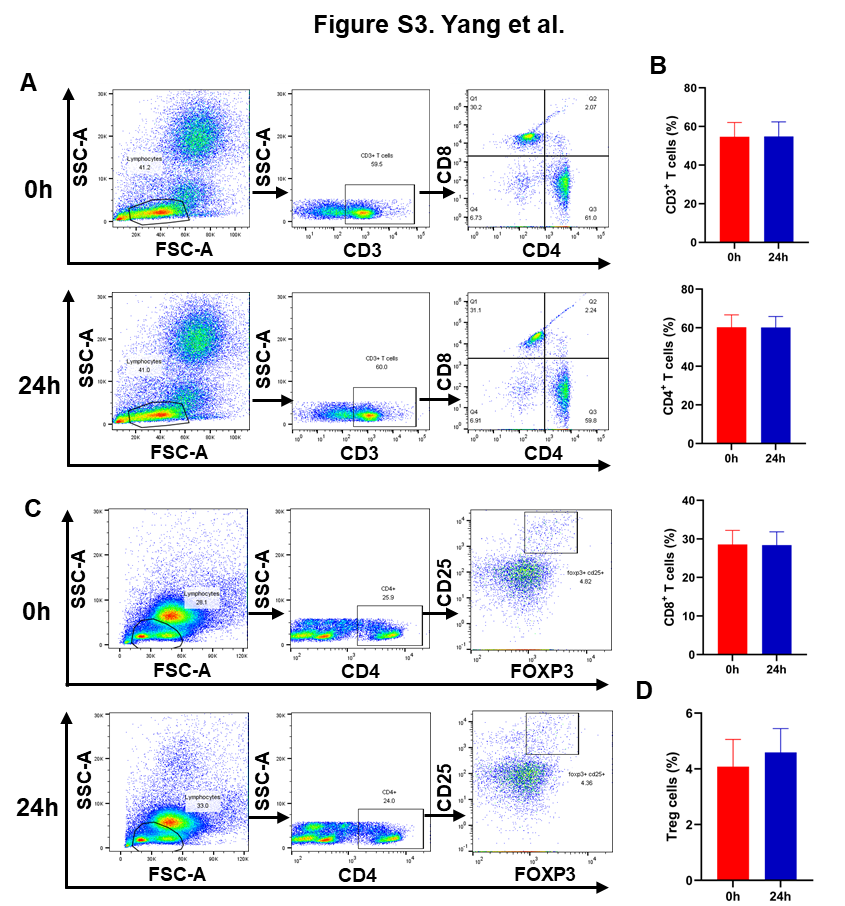


Figure S3. Flow cytometry analysis of CD3^+^ T cells, CD4^+^ T cells, CD8^+^ T cells and Treg cells in peripheral blood of rats after hUC-MSC transplantation. (A) Flow cytometry analysis of CD3^+^ T cells, CD4^+^ T cells, and CD8^+^ T cells in peripheral blood in rats at 24 hours after hUC-MSC injection. (B) Quantification of the percentages of CD3^+^ T cells, CD4^+^ T cells, and CD8^+^ T cells in peripheral blood of rats and the data are presented as mean ± SD (n=3). (C) Flow cytometry analysis of T CD4^+^CD25^+^FOXP3^+^ T (Treg) cells in peripheral blood of rats at 24 hours after hUC-MSC injection. (D) Quantification of the percentages of Treg cells in peripheral blood of rats and the data are presented as mean ± SD (n=3).


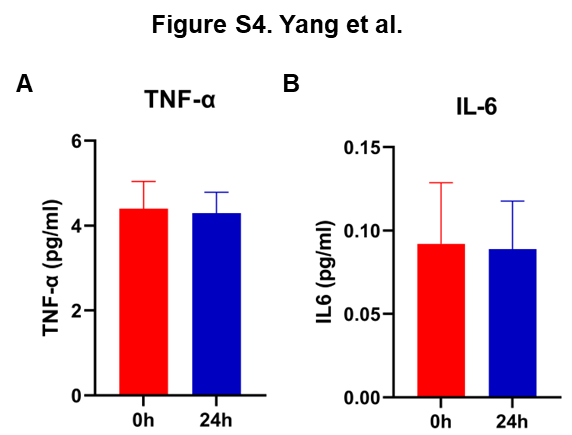


Figure S4. The concentration of TNF-α and IL-6 in peripheral blood of rats at 24 hours after hUC-MSC transplantation detected by ELISA.

| Antibodies | Company | Catalogue number | Host species | concentrations | Dilution |
| --- | --- | --- | --- | --- | --- |
| Anti-nNOS antibody | Abcam | ab5586 | Rabbit | 1 mg/ml | 1:400 |
| Anti-eNOS antibody | Abcam | ab5589 | Rabbit | 0.2 mg/ml | 1:400 |
| Anti-desmin antibody | Abcam | ab32575 | Rabbit | 0.025 mg/ml | 1:400 |
| Anti-α-SMA antibody | CST | #19245 | Rabbit | 7 µg/ml | 1:400 |
| Anti-SIRT1 antibody | Abcam | ab189494 | Rabbit | 0.52 mg/ml | 1:1000 |
| Anti-PGC-1α antibody | Novus | NBP1-04676 | Rabbit | 1 mg/ml | 1:1000  (Western Blotting)  1:100  (Immunoprecipitation) |
| Acetylated-Lysine Antibody | CST | #9441 | Rabbit | 663 µg/ml | 1:1000 |
| Anti-TFAM antibody | Abclonal | A3173 | Rabbit | 1mg/ml | 1:1000 |
| Anti-NDUFB8 antibody | ProteinTech | 14794-1-AP | Rabbit | 650 μg/ml | 1:1000 |
| Anti-SDHB antibody | ProteinTech | 10620-1-AP | Rabbit | 600 μg/ml | 1:1000 |
| Anti-UQCRC2 antibody | ProteinTech | 14742-1-AP | Rabbit | 800 ug/ml | 1:1000 |
| Anti-MTCO2 antibody | ProteinTech | 55070-1-AP | Rabbit | 500 μg/ml | 1:1000 |
| Anti-ATP5A antibody | ProteinTech | 14676-1-AP | Rabbit | 400 μg/ml | 1:1000 |
| Anti-TOM20 antibody | Abcam | ab56783 | Mouse | 1 mg/ml | 1:1000 |
| Anti-Cleaved caspase3 antibody | Abclonal | A11021 | Rabbit | 500 μg/ml | 1:1000 |
| Anti-Caspase3 antibody | Abclonal | A0214 | Rabbit | 500 μg/ml | 1:1000 |
| Anti-Bcl2 antibody | Abclonal | A19693 | Rabbit | 500 μg/ml | 1:1000 |
| Anti-β-Actin antibody | ProteinTech | 66009-1-Ig | Mouse | 1 mg/ml | 1:5000 |
| HRP-conjugated anti-mouse secondary antibody | ProteinTech | SA00001-1 | Goat | 0.2 mg/ml | 1:10000 |
| HRP-conjugated anti-rabbit secondary antibody | ProteinTech | SA00001-2 | Goat | 0.2 mg/ml | 1:10000 |

**Table S1. The information of primary antibodies used in the study.**
