# Supplementary material for: hUC-MSC preserves erectile function by restoring mitochondrial mass of penile smooth muscle cells in a rat model of cavernous nerve injury via SIRT1/PGC-1a/TFAM signaling
Source: Biol Res. 2025 Jan 27;58:8. doi: 10.1186/s40659-024-00578-y (PMC11773750; doi:10.1186/s40659-024-00578-y)
Supplement: Supplementary file 2 — Additional file 2 [file 40659_2024_578_MOESM2_ESM.pdf]

Original western blots

Antibody Validation

NDUFB8 22KD

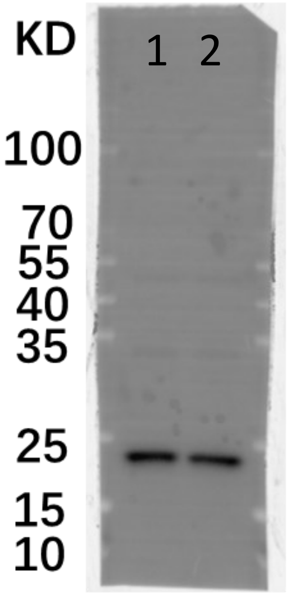

Line1:rat heart tissue

Line2:rat heart tissue

Two independent sample

SDHB 32KD

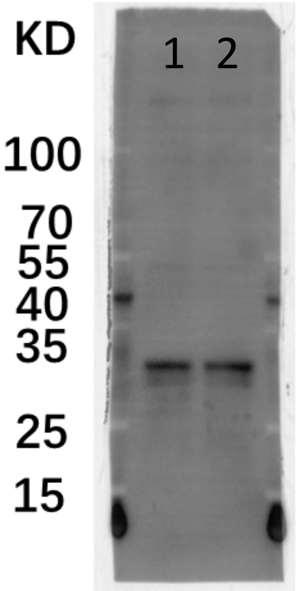

Line1:rat heart tissue

Line2:rat heart tissue

Two independent sample

UQCRC2 48KD

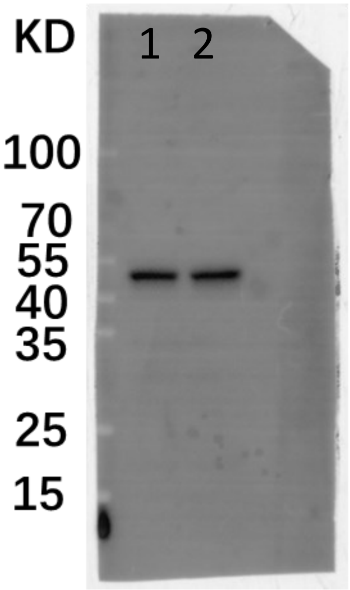

Line1:rat heart tissue

Line2:rat heart tissue

Two independent sample

MTCO2 26KD

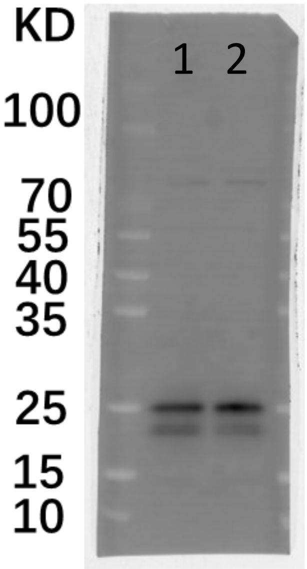

Line1:rat heart tissue

Line2:rat heart tissue

Two independent sample

ATP5A1 60KD

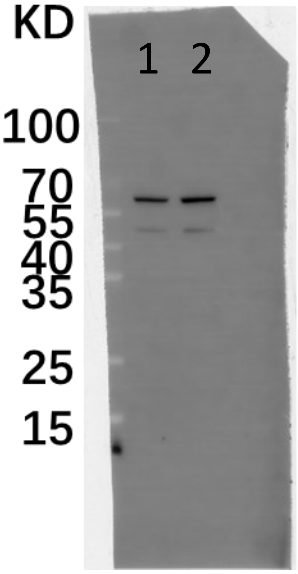

Line1:rat heart tissue

Line2:rat heart tissue

Two independent sample

SIRT1 110KD

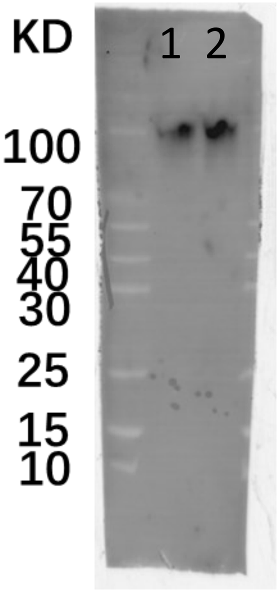

Line1:rat testi tissue

Line2:rat testi tissue

Two independent sample

Original western blots

Antibody Validation

PGC-1α 100KD

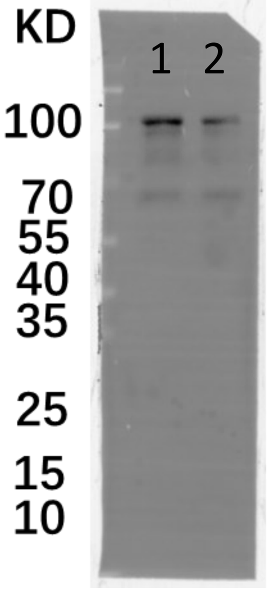

Line1:rat heart tissue

Line2:rat heart tissue

Two independent sample

TFAM 26KD

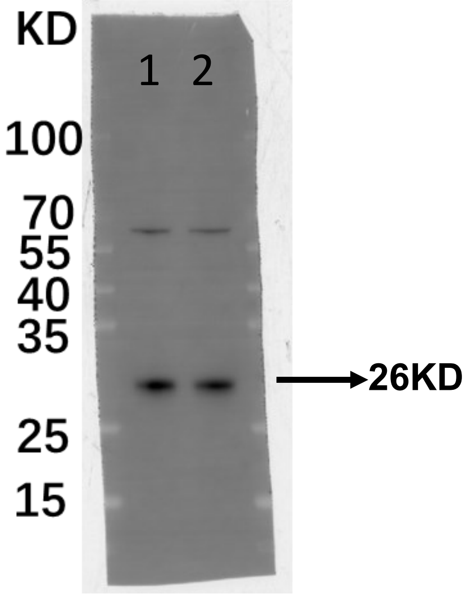

Line1:rat heart tissue

Line2:rat heart tissue

Two independent sample

Cleaved caspase3 17KD

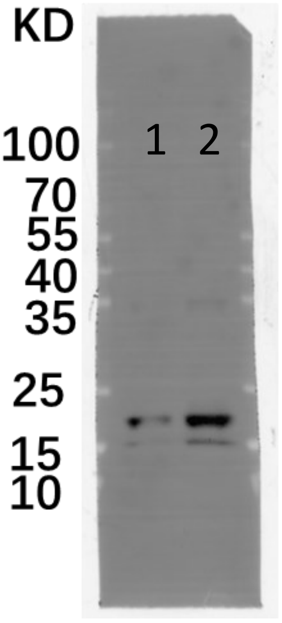

Line1:100μM H_2O_2 treated schwan cells

Line2:200μM H_2O_2 treated schwan cells

Bcl2 26KD

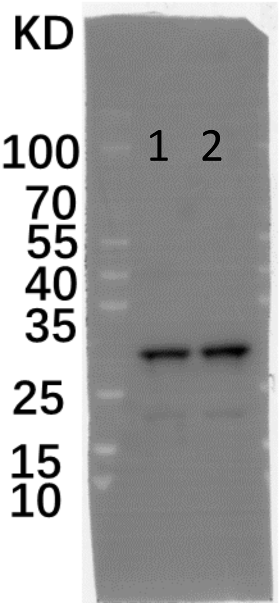

Line1:schwan cells

Line2:schwan cells

Original western blots

Figure S1

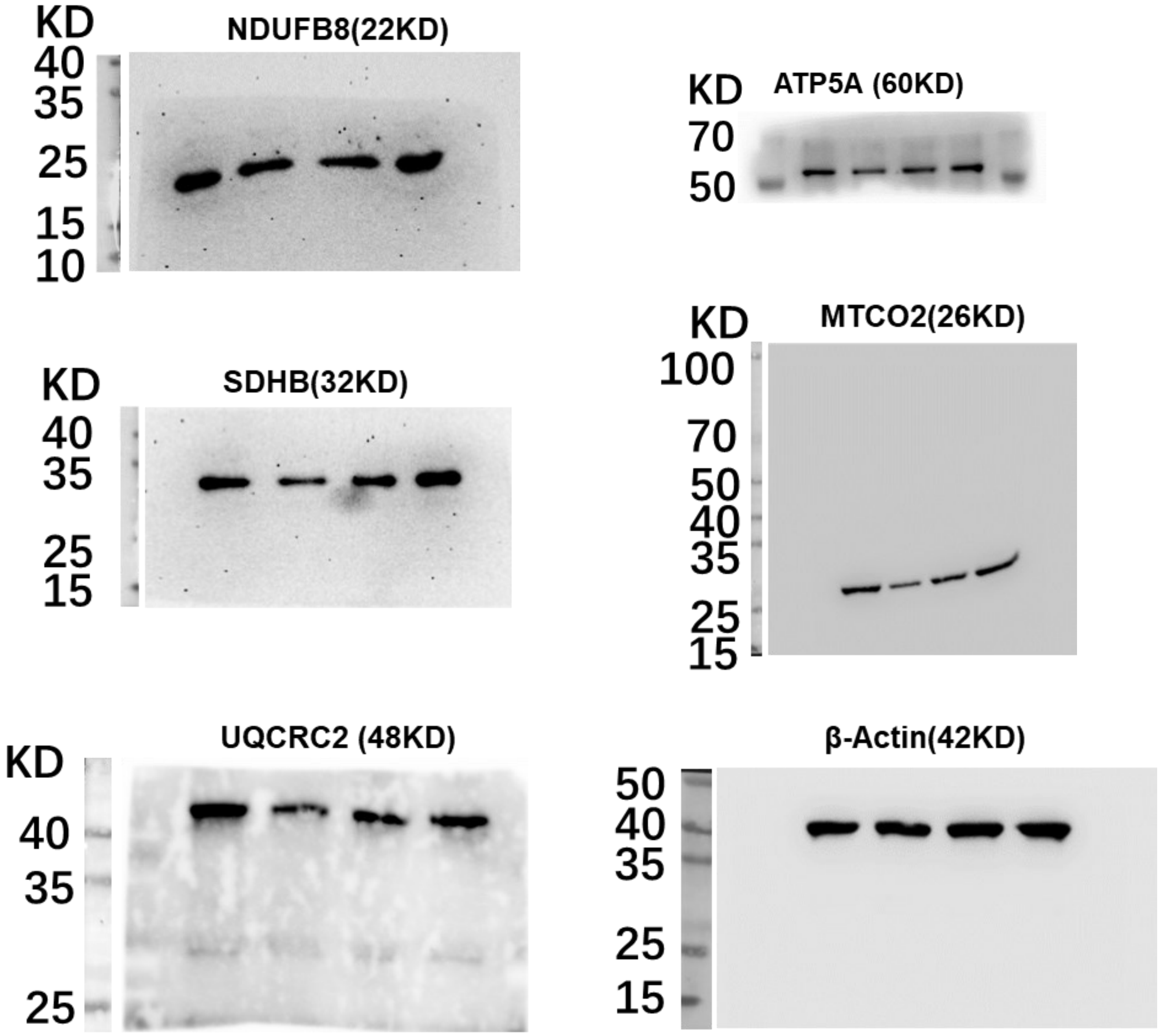

Figure S2

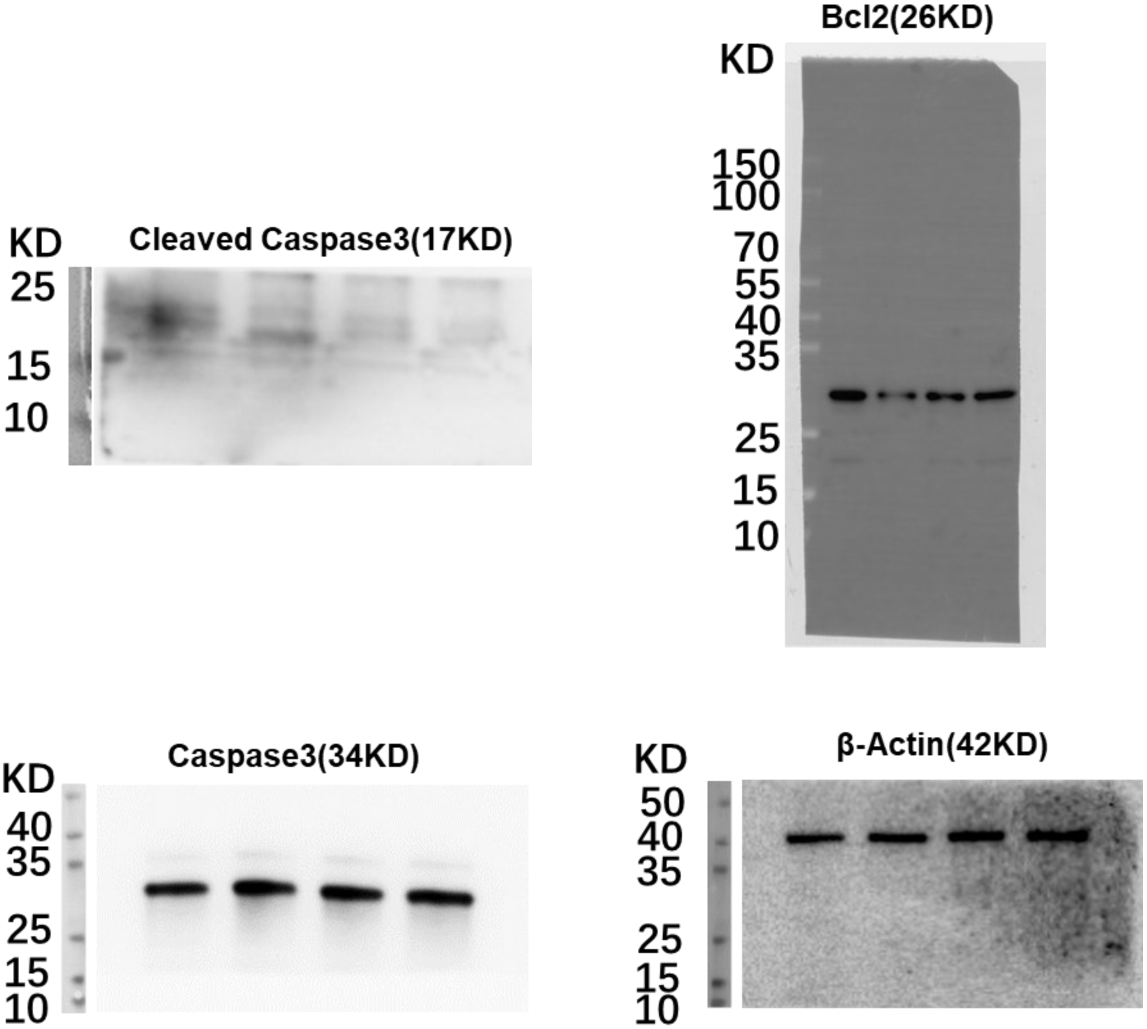
Cleaved Caspase3(17KD)
KD
25
15
10
Bcl2(26KD)
KD
150
100
70
55
40
35
25
15
10
Caspase3(34KD)
KD
40
35
25
15
10
β-Actin(42KD)
KD
50
40
35
25
15
10

Figure S3

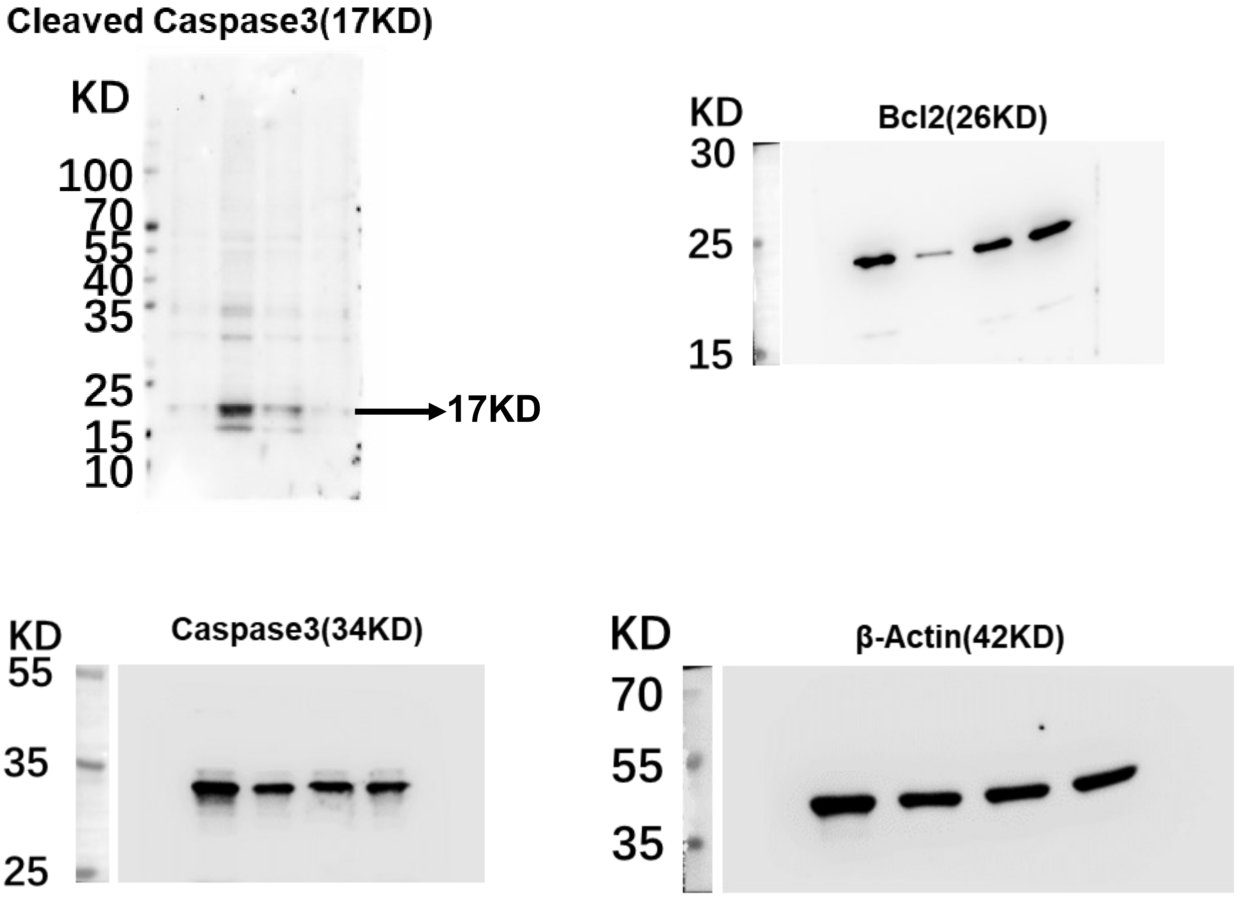

Cleaved Caspase3(17KD)
KD
100
70
55
40
35
25
15
10
17KD
KD
Bcl2(26KD)
30
25
15
KD
Caspase3(34KD)
55
35
25
KD
β-Actin(42KD)
70
55
35

Figure S4

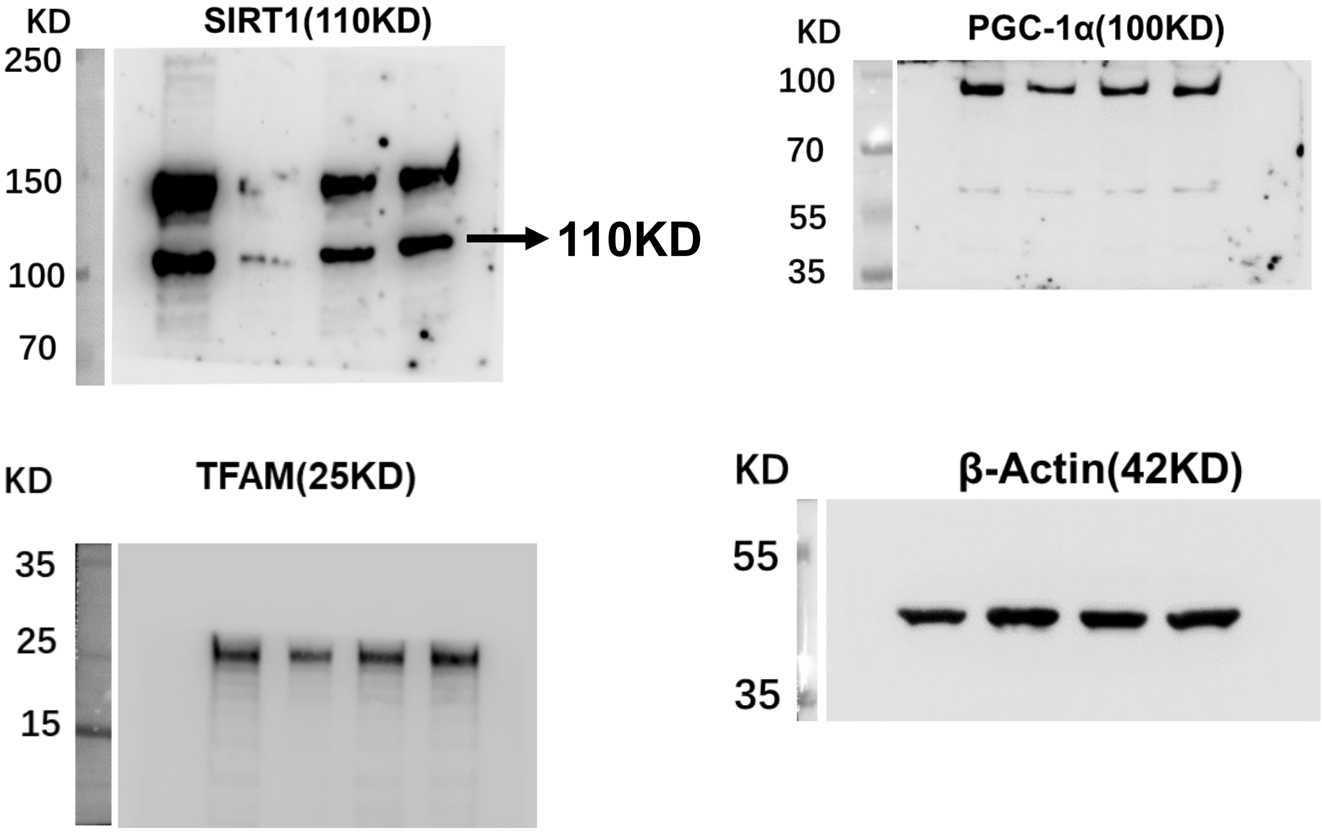

KD
SIRT1(110KD)
250
150
100
70
110KD
KD
PGC-1α(100KD)
100
70
55
35
KD
TFAM(25KD)
35
25
15
KD
β-Actin(42KD)
55
35

Figure S5

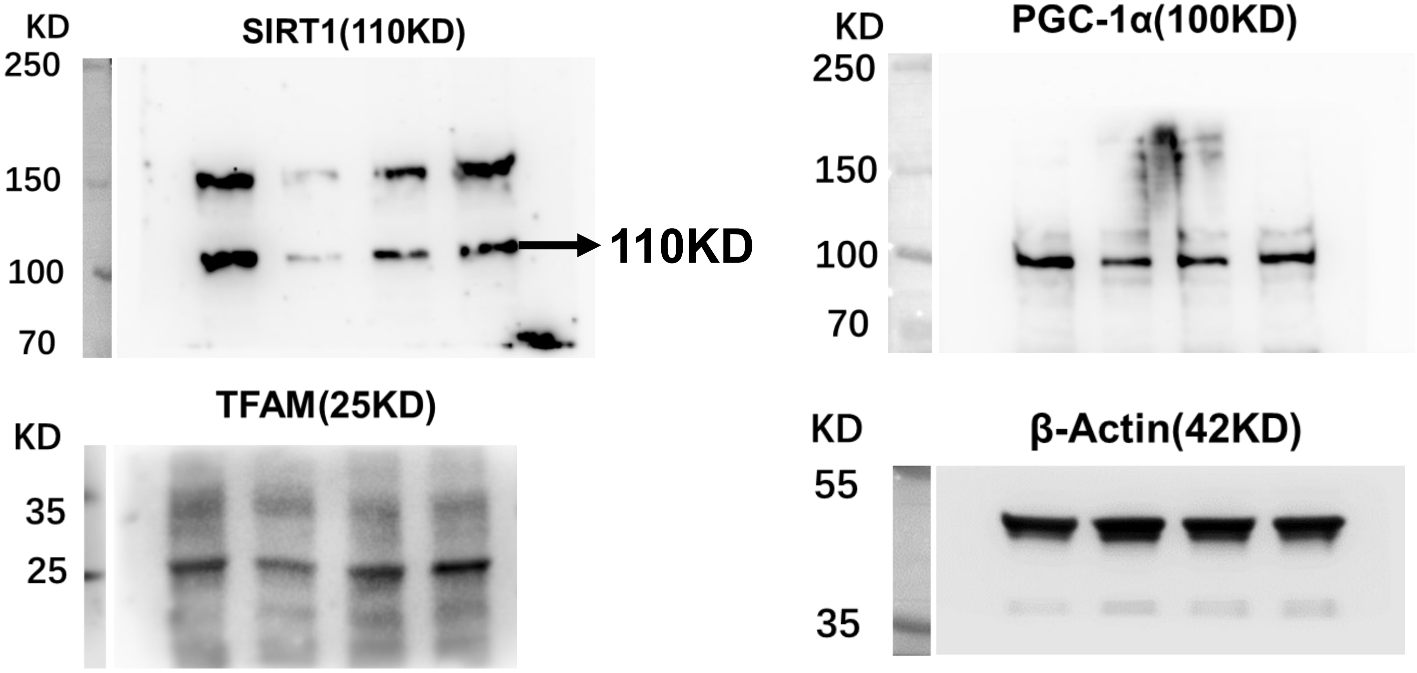

Figure S6

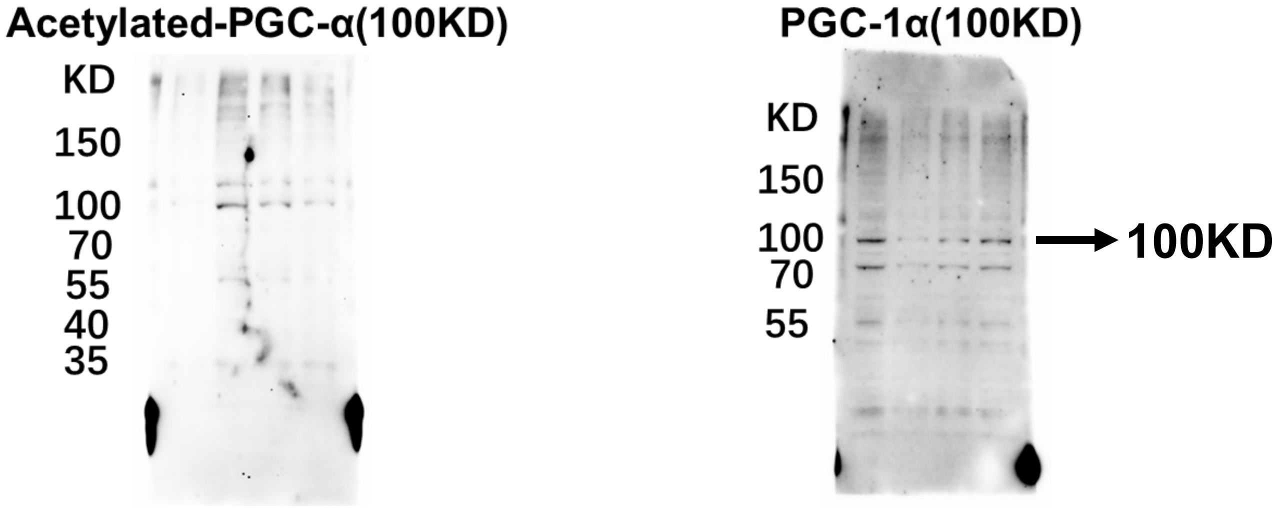

Figure S7

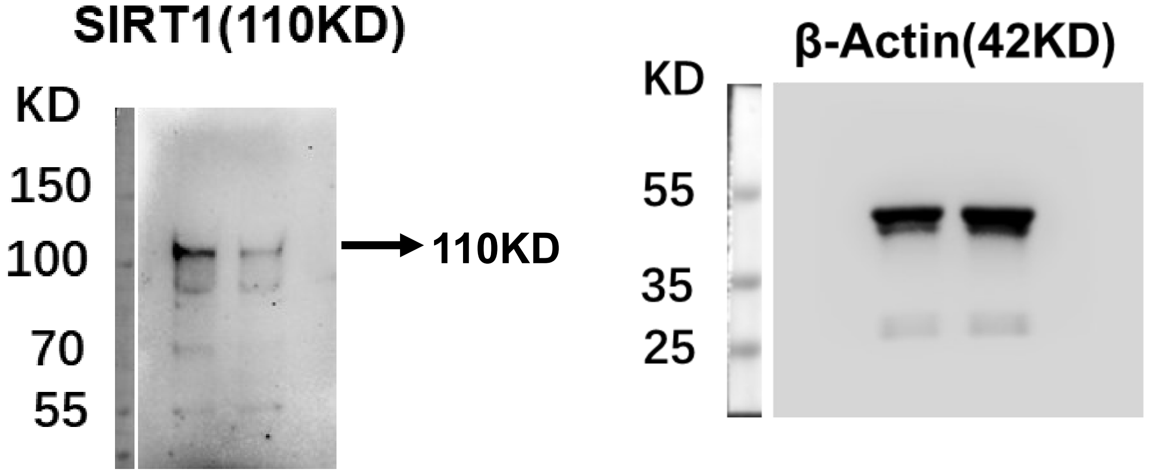

Figure S8

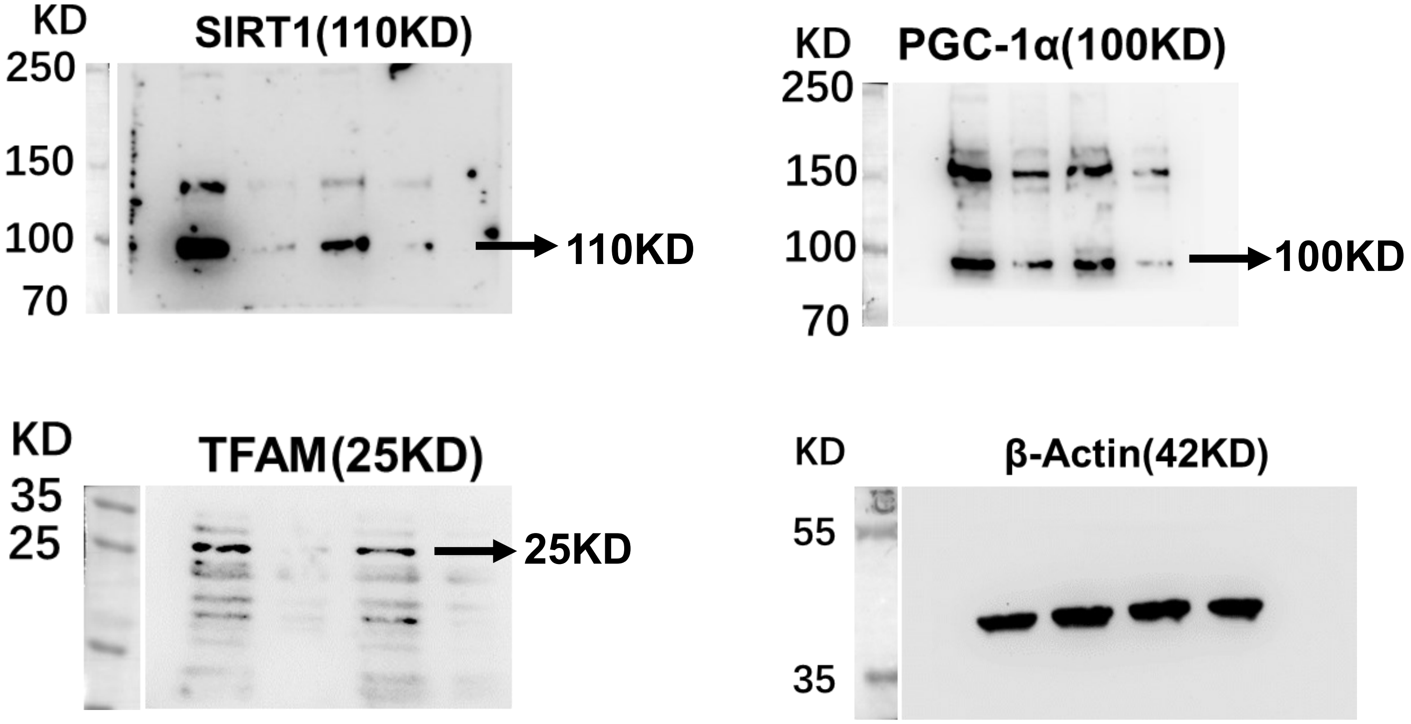

Figure S9

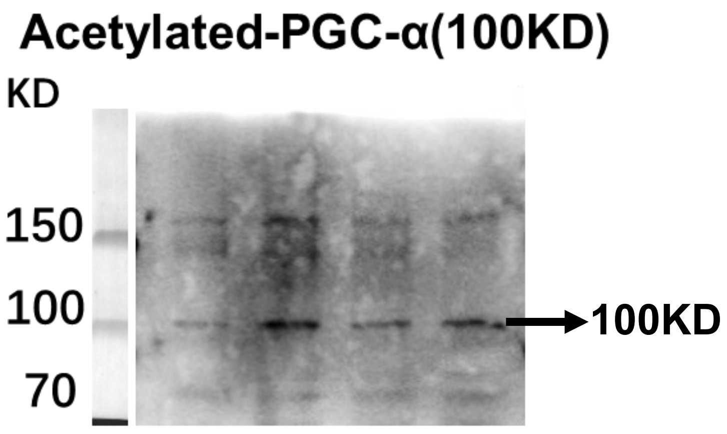

Acetylated-PGC-α(100KD)
KD
150
100
70
100KD

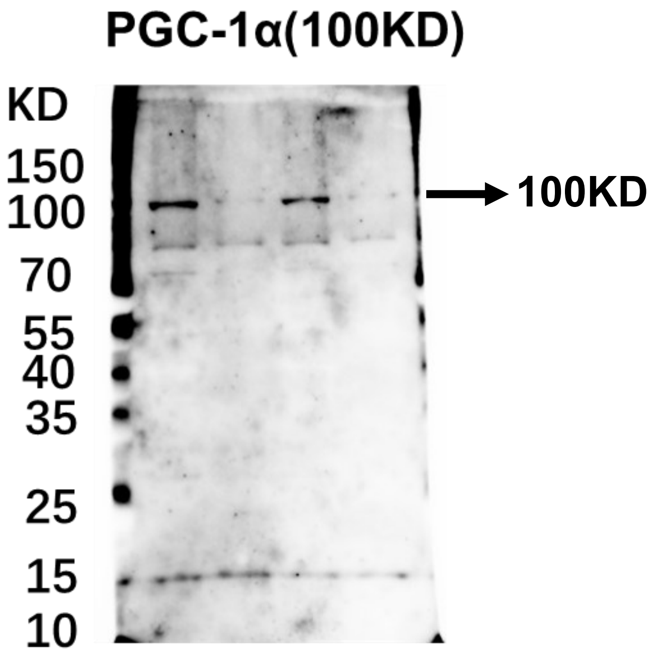

PGC-1α(100KD)
KD
150
100
70
55
40
35
25
15
10
100KD

Figure S10

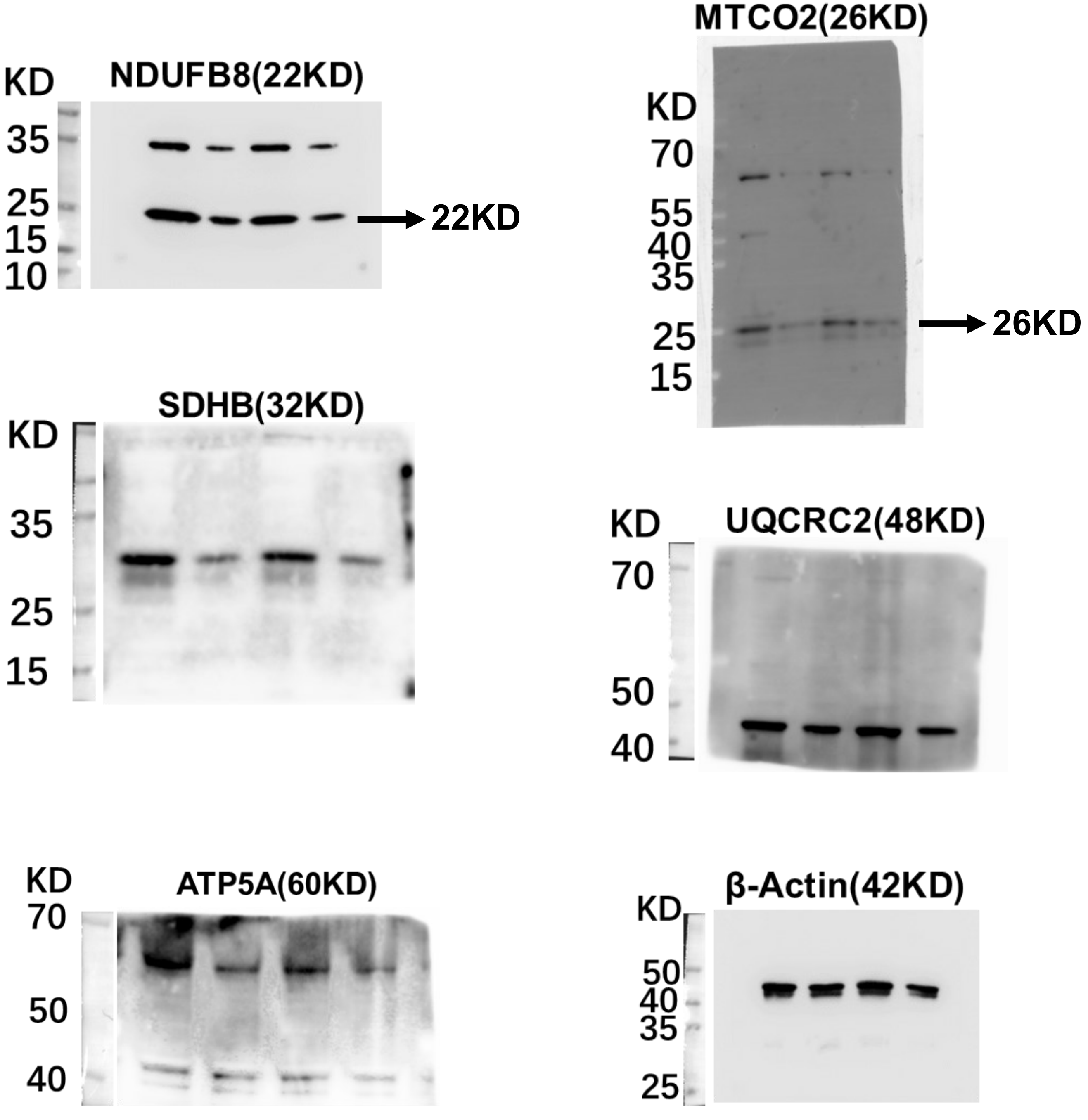
NDUFB8(22KD)
KD
35
25
15
10
22KD
MTCO2(26KD)
KD
70
55
40
35
25
15
26KD
SDHB(32KD)
KD
35
25
15
UQCRC2(48KD)
KD
70
50
40
ATP5A(60KD)
KD
70
50
40
β-Actin(42KD)
KD
50
40
35
25

Figure S11

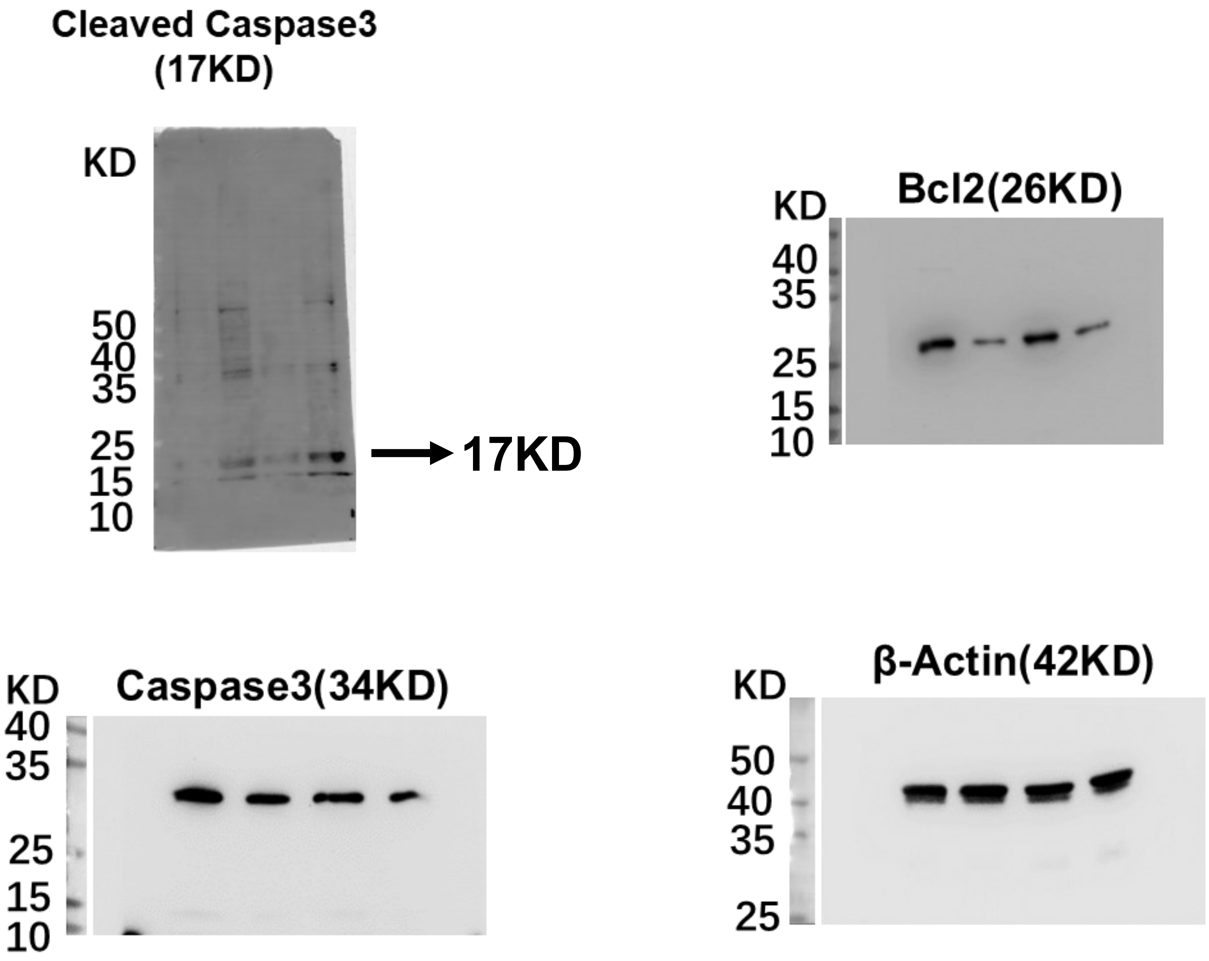
Cleaved Caspase3 (17KD)
KD
50
40
35
25
15
10
17KD
Bcl2(26KD)
KD
40
35
25
15
10
Caspase3(34KD)
KD
40
35
25
15
10
β-Actin(42KD)
KD
50
40
35
25
